# Persulfate assisted photocatalytic and antibacterial activity of TiO_2_–CuO coupled with graphene oxide and reduced graphene oxide

**DOI:** 10.1038/s41598-024-63452-7

**Published:** 2024-05-31

**Authors:** Charitha Thambiliyagodage, Heshan Liyanaarachchi, Madara Jayanetti, Geethma Ekanayake, Amavin Mendis, Upeka Samarakoon, Saravanamuthu Vigneswaran

**Affiliations:** 1 Faculty of Humanities and Sciences, Sri Lanka Institute of Information Technology, Malabe, Sri Lanka; 2 Department of Nano Science Technology, Faculty of Technology, Wayamba University of Sri Lanka, Kuliyapitiya, Sri Lanka; 3 Faculty of Engineering and Information Technology, University of Technology Sydney, PO Box 123, Broadway, NSW 2007 Australia; 4 Faculty of Sciences and Technology (RealTek), Norwegian University of Life Sciences, P.O. Box 1432, Ås, Norway

**Keywords:** Chemistry, Materials science, Nanoscience and technology

## Abstract

Photocatalysts of TiO_2_–CuO coupled with 30% graphene oxide (GO) were hydrothermally fabricated, which varied the TiO_2_ to CuO weight ratios to 1:4, 1:2, 1:1, 2:1 and 4:1 and reduced to form TiO_2_–CuO/reduced graphene oxide (rGO) photocatalysts. They were characterized using XRD, TEM, SEM, XPS, Raman, and DRS technologies. TiO_2_–CuO composites and TiO_2_–CuO/GO degrade methylene blue when persulfate ions are present. Persulfate concentration ranged from 1, 2, 4 to 8 mmol/dm^−3^ in which the highest activity of 4.4 × 10^–2^ and 7.35 × 10^–2^ min^−1^ was obtained with 4 mmol/dm^−3^ for TiO_2_–CuO (1:4) and TiO_2_–CuO/GO (1:1), respectively. The presence of EDTA and isopropyl alcohol reduced the photodegradation. TiO_2_–CuO coupled with rGO coagulates methylene blue in the presence of persulfate ions and such coagulation is independent of light. The catalyst dosage and the concentration of the dye were varied for the best-performing samples. The antibacterial activity of the synthesized samples was evaluated against the growth of *Escherichia coli*, *Staphylococcus aureus*, *Pseudomonas aeruginosa* and *Klebsiella pneumonia*. Ti:Cu (1:2)-GO and Ti:Cu (1:4)-GO had the highest antibacterial activity against *K*. *pneumoniae* (16.08 ± 0.14 mm), *P*.* aeruginosa* (22.33 ± 0.58 mm), *E*. *coli* (16.17 ± 0.29 mm) and *S*. *aureus* (16.08 ± 0.88).

## Introduction

Rapid industrialization has led to much damage being done to the environment. Synthetic dyes in the textile industry have contributed much in the way of pollution, leading to the extensive poisoning and deaths of aquatic habitats and organisms^1–3^. The process of textile dyeing aims to produce colored fabric with a desired shade, homogeneous hue, depth of shade, and satisfactory color fastness properties^4^. Synthetically produced dyes are used on an industrial scale due to their versatility and economic advantages over other dyes^5^. Azo dyes are the cheapest to make, have the highest intensity, and the best color fastness of all synthetic dyes and account for 60% of dyes used in industry^6–8^. Azo dyes pose an immense risk to the environment when released into it. This is mainly due to their primary byproduct, aromatic amines which arise due to the cleavage of the azo bond. These byproducts are classified as carcinogens, and they are dangerous to human health^9^. Aquatic organisms and their habitats are seriously affected by the release of dyes into waterways, resulting in the destruction of aquatic ecosystems^10^. Vegetation and crops can be destroyed by azo dyes as well due to their phytotoxic effects^11^.

Currently, biological degradation is the most widespread technique utilizing aerobic and anaerobic digestive systems^12^. Adsorbent materials such as activated carbon and silica have also been employed to remove dyestuff in smaller concentrations, yet the technique is limited by its capacity and reusability^13,14^. Flocculation using agents such as ferric (Fe^3+^) or aluminum (Al^3+^) ions have been deployed in dye removal^15^. Chlorine and ozone to promote chemical degradation of dye effluents have emerged recently but they are limited by the costs associated with the oxidizing agents used^16^. The major focus has been on devising novel techniques for the removal and treatment of industrial dyes before they are released into normal water reservoirs.

Use of nanoparticles for the treatment of pollutants has been offered as a new solution for this long-standing issue^14^. In particular, inorganic nanophotocatalysts have proven to be efficient in degrading persistent organic dyes^17^. Energy of light in the visible spectrum is harnessed in generating radicals, which effectively degrades the dye molecules present in the effluent. Novel heterogenous photocatalytic systems have been investigated in order to increase the rate and efficiency of digestion of these dyes^18–22^. These catalysts are advantageous in comparison to other methods due to their reusability, amount of the catalyst required for treatment and complete degradation of the pollutant molecules. In addition to generating hydroxyl (·OH) and superoxide (O_2_·^−^) radicals, supplementing the photocatalysts with persulfates [peroxydisulfate (S_2_O_8_^2−^) and peroxymonosulfate (HSO_5_^−^) can lead to the generation of sulphate radicals (SO_4_·^−^) which otherwise cannot be generated by the catalyst under visible light^23^. The increased number of radicals will result in better degradation of the dye molecules. Titanium dioxide or titania (TiO_2_) serves as a better photocatalyst due to the stability of the compound, high quantum yield and non-toxicity^24^. However, the activity of titania is mainly limited to the UV range of the electromagnetic spectrum due to its band gap value of 3.0 eV^25^. Cupric oxide (CuO), due to its properties such as large surface areas, proper redox potential, good electrochemical activity superthermal conductivity, and excellent stability in solutions, becomes an interesting candidate for further exploration^26^. CuO nanoparticles possessing a wide absorption band in the visible region can lead to the production of electrons and holes under visible light irradiation^17,27^. Modification of titania by incorporating metals and non-metals has recently become a focus of research due to the preexisting photocatalytic properties which can be enhanced and expanded^28–33^. CuO is an excellent complement in creating an efficient heterogenous catalyst capable of increasing the activity and stability of the degradation process. Recently, photocatalysts have been immobilized on frameworks such as GO, g-C_3_N_4_ which further enhances the photocatalytic activity of the metals or the metal oxides. Mo doped ZnO nanoparticles loaded on g-C_3_N_4_ nanosheets^34^, transition metals like Fe, Co, Ni, Mn and Cr doped ZnO coupled g-C_3_N_4_^35^, Cd doped ZnO supported on g-C_3_N_4_^36^, SnO_2_/ZnO coupled with GO^37^, MgO coupled with GO^38^ have shown to effective in degrading pollutants such as methylene blue, rhodamine B, reactive blue 222, sulfamethazine, atrazine.

In our study, we investigate the effective photodegradation of commonly used azo dye methylene blue with the use of TiO_2_/CuO photocatalyst combined with graphene oxide and reduced graphene oxide hydrothermally. It has the ability to increase the conductance of electrons by incorporating a graphene framework which improves the charge carrier separation. The photodegradation efficiency of the catalyst was supplemented using persulphate. The supplemented heterogenous inorganic catalyst system performed well by efficiently digesting the methylene blue molecules under LED lights in the visible range. Further, the reusability of the photo catalyst, effects of dye concentration and effective dosages of the catalyst were explored to assess the efficient and practical application in an industrial setting.

## Materials and methodology

### Chemicals and materials

TiO_2_ (P25), CuSO_4_, H_2_SO_4_ (99.8%), H_3_PO_4_ (37.5%), and H_2_O_2_ (30%) were procured from Sigma Aldrich (UK). NaOH pellets, NaBH_4,_ and KMnO_4_ were purchased from Sisco Research Laboratories (Pvt) Ltd, India. Methylene blue (98%), Muller Hinton Agar (MHA) and Luria Bertani Broth (LB broth) were purchased from HiMedia Leading Biosciences Company (Maharashtra, India). Graphite was obtained from Bogala, Sri Lanka. Deionized water (DI), with resistivity greater than 18.0 MΩ cm (Millipore Milli-Q system), was used in the experiments. All the chemicals utilized were of analytical grade and utilized without further purification.

### Synthesis of the nanocomposites

The nanocomposites were synthesized via coprecipitation, in different ratios of TiO_2_ and CuO as follows: TiO_2_:CuO, 1:1, 1:2, 1:4, 2:1, 4:1. TiO_2_ (P25) was dispersed in 10 M NaOH and hydrothermally treated at 180 °C for 24 h. CuSO_4_ was dissolved in a minimal amount of deionized water and an adequate amount of 1 M NaOH was added and stirred until the CuO precipitate was obtained. The obtained sample was then filtered and washed with deionized water until the samples were free of SO_4_^2−^ ions, and a neutral pH was achieved. Both hydrothermally treated TiO_2_ and obtained CuO were mixed and sonicated for 2 h. Following this the mixtures were hydrothermally treated in 10 M NaOH medium at 180 °C for 15 h. The washed samples were then oven-dried at 100 °C until completely dried and stored for further analysis.

### Synthesis of graphene oxide and reduced graphene oxide

Optimized Hummer’s method served to synthesize graphene oxide (GO) using natural graphite powder. Graphite powder and KMnO_4_ at a 1:3 ratio was homogenized together via mechanical grinding. The required acid combination was prepared by mixing 360 mL of conc. H_2_SO_4_ (99.8%) and 40 mL of conc. H_3_PO_4_ (37.5%) at a ratio of 9:1 and maintained at a lower temperature in an ice bath to stop the exothermic reaction that takes place when the graphite and KMnO_4_ combination is added to the acid mixture. The powder was treated with the acid mixture and the resulting dark yellowish-green solution was stirred for 24 h at 55 °C. After allowing the liquid to reach room temperature, 3 mL of 30% hydrogen peroxide (H_2_O_2_) was added, and stirring was done for 5 min. The next exothermic reaction was regulated by ice cubes, and the resultant solution was yellowish-orange in color. After obtaining the solid, it was rinsed with deionized water until the pH reached neutral and negative for SO_4_^2−^ ions. Finally, the gathered material was dried for one hour at 60 °C, and in this paper, it is referred to as GO. Synthesized graphene oxide was treated with sodium borohydride (NaBH_4_) to obtain reduced graphene oxide. It is referred to as rGO in this study.

### Fabrication of the coated nanocomposites

The TiO_2_/CuO composites in different ratios were coupled with GO constituting 30% of the total weight hydrothermally at 150 °C for 18 h. NaBH_4_ was added to TiO_2_/CuO/GO samples to synthesize TiO_2_/CuO/rGO composites. The synthesized composites coupled with graphene oxide, and reduced graphene oxide are expressed as TC (1:1), TC (1:2), TC (1:4), TC (2:1), TC (4:1), TCG (1:1), TCG (1:2), TCG (1:4), TCG (2:1), TCG (4:1), TCrG (1:1), TCrG (1:2), TCrG (1:4), TCrG (2:1), TCrG (4:1) for the purposes of this research.

### Antibacterial activity

#### Preparation of media

The necessary amounts of growth culture media were prepared by mixing Muller Hinton agar and Luria Bertani broth with deionized water, followed by complete sterilization in the autoclave.

#### Microbial strain and inoculum preparation

The gram-negative *E*.* coli*, *P*.* aeruginosa*, *K*.* pneumoniae*, and gram-positive *S*.* aureus*, functioning as the test organisms, were sourced from the Medical Research Institute, Sri Lanka. Cultures of *E*.* coli*, *S*.* aureus*, *P*.* aeruginosa*, and *K*.* pneumoniae* were introduced to the Luria Bertani broth medium and incubated at 37 °C to prepare the inocula. The microbial cultures were sub-cultured and subsequently allowed to grow for 24 h prior to the assay. The concentrations were then adjusted and diluted to attain a microbial suspension containing 5 × 10^5^ colony-forming units (CFUs)/mL using a UV–visible spectrophotometer for subsequent analysis^39^.

#### Agar well diffusion method

Nanomaterials were weighed (20 and 40 mg) and sonicated in Dimethyl sulfoxide (DMSO) for 1 h. The Mueller Hinton Agar plate was inoculated by spreading the adjusted microbial inoculum of 5 × 10^5^ colony-forming units (CFUs)/mL over the entire agar surface via streaking. Holes were punched aseptically with a sterile cork borer, and 70 μL of the antimicrobial agent solution of desired concentrations—20, or 40 mg in 1 mL of DMSO—were introduced into the wells. A standard antibiotic (amoxicillin) serving as the positive control and Dimethyl sulfoxide (DMSO) as the negative control were also introduced into one well each. Three replicates for each sample and each species of bacteria were prepared. The prepared agar plates were then incubated for about 18 h at 37 °C exposing to LED light and the zones of inhibition were measured in mm.

### Photocatalytic activity

The photocatalytic activity of the synthesized nanocomposites was persulphate assisted and evaluated for the degradation of the methylene blue dye under LED light. In the experiment, 25 mg of the nanocomposite was shaken in 25 mL of a 25 mg/L methylene blue aqueous solution for 18 h to reach the adsorption–desorption equilibrium. Then the solution was decanted and resuspended in 50 mL of 10 mg/L methylene blue solution and kept in the dark for 30 min. The samples were subsequently exposed to normal LED light, and during that period, aliquots were withdrawn at 15-min intervals, and the samples were analyzed by taking absorbance readings using a UV–visible spectrophotometer. Persulfate concentration ranged from 1, 2, 4, and 8 mM for the purposes of investigating the impact of the concentration of persulfate on photocatalysis. The effect of scavengers IPA (4 mM) and EDTA (4 mM) were added just before exposing the solutions to the light source in the presence of 4 mM of persulfate ions to study the effect of scavengers. The weight of the catalyst and the concentration of dye (MB) were also varied in the presence of 4 mM of persulfate in the medium.

## Material characterization

X-Ray diffraction analysis (XRD) patterns were collected using the D8 Advance Bruker system, with Cu K α (λ = 0.154 nm) anode, varying the 2θ from 5° to 80° at a scan speed of 2°/min. The scanning electron microscopic (SEM) images were acquired using a Carl ZEISS EVO 18 RESEARCH instrument. A transmission electron microscope (TEM) (JEOL–JEM-2100) operating with 200 kV served to characterize the morphology of the synthesized nanocomposites. 1 µL of nanomaterial dispersed in ethanol was mounted on a carbon copper grid with holes and allowed to dry at room temperature before the TEM analysis commenced. The surface chemistry of the nanomaterials was analyzed by X-ray photoelectron spectroscopy (XPS). The Thermo Scientific ESCALAB Xi + X-ray photoelectron spectrometer acquired the survey spectra and higher-resolution spectra of the synthesized catalysts. To carry out the Raman analysis, a Bruker Senterra Raman microscope spectrophotometer was used. The diffuse reflectance spectra (DRS) of the powder samples were analyzed using a Shimadzu 1800 UV/Visible spectrophotometer armed with a precision Czerny-Turner optical system. Measurements were done using a bandwidth of 1.0 nm and a wavelength range of 400–750 nm. The absorbance of the methylene blue samples was measured using a Shimadzu UV-1990 double-beam UV–visible spectrophotometer.

## Results and discussion

### XRD

**Figure 1:**
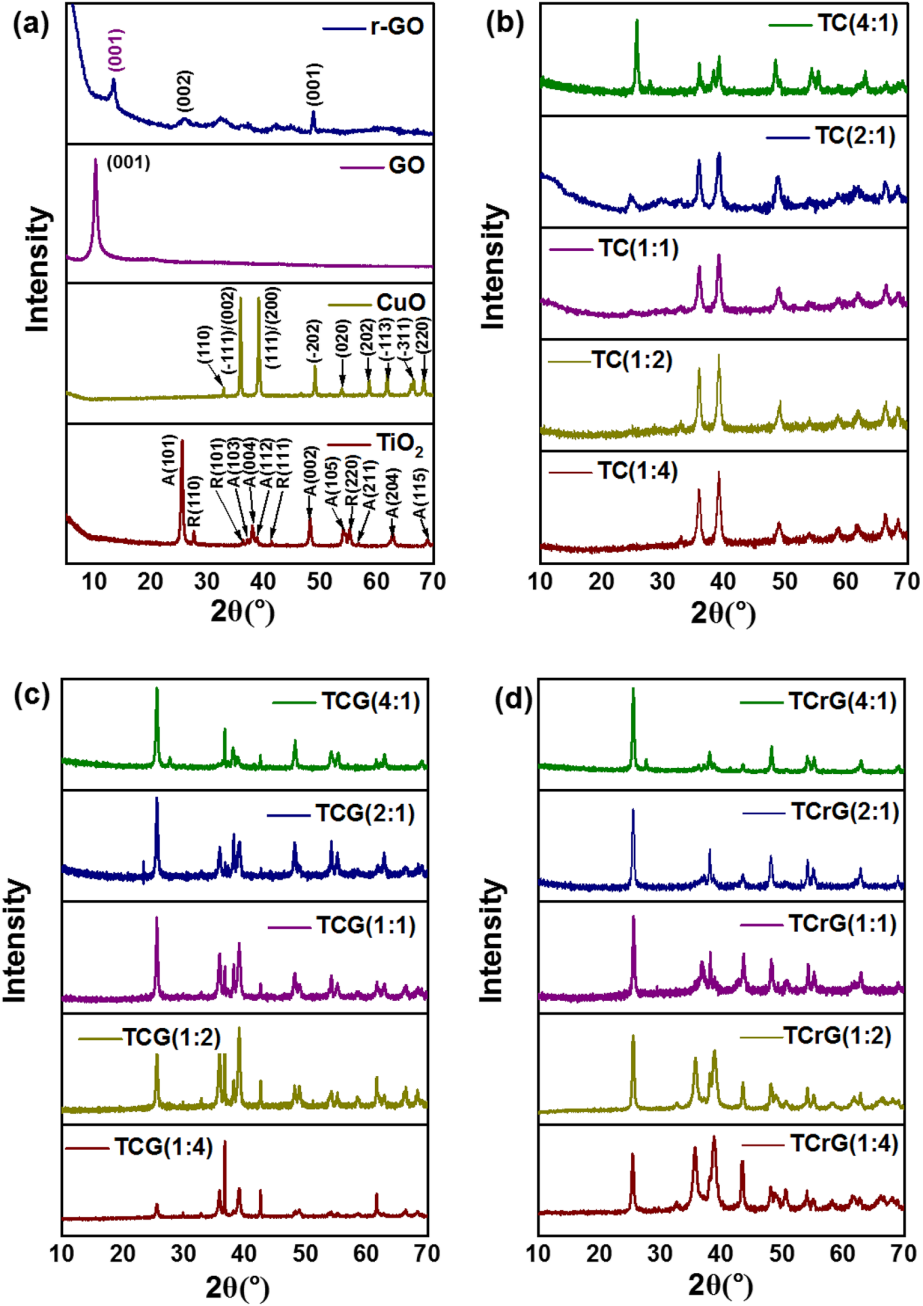
XRD patterns of (**a**) TiO_2_, CuO, GO, and rGO (**b**) TC composites (**c**) TCG composites (**d**) TCrG composites.

The XRD patterns identified the crystal nature and phase purity of the synthesized nanocomposites (Fig. 1). The XRD pattern of pure P25 TiO_2_ shows peaks at 25.42°, 27.61°, 36.30°, 37.10°, 37.94°, 38.77°, 41.37°, 48.17°, 54.09°, 55.19°, 56.76°, 62.89, and 69.18°. These correspond respectively to the crystalline planes of A(101), R(110), R(101), A(103), A(004), A(112), R(111), A(002), A(105), R(220), A(211), A(204) and A(115), where A stands for Anatase phase and R denotes the Rutile phase. The interlayer distance, crystalline size and number of crystallites of Anatase phase are 0.35 nm, 46 and 131.42, respectively, and those of Rutile phase are 0.32 nm, 90 and 56.7, respectively. The (110), (− 111)/(002), (111)/(200), (− 202), (020), (202), (− 113), (− 311), and (220) crystalline planes are represented by the peaks at 32.84°, 35.55°, 35.57°, 38.41°, 38.73°, 48.64°, 53.30°, 61.75°, 66.38°, 68.40°, 72.40°, and 75.16° in the XRD pattern of CuO with 0.23 nm of interlayer distance, 64.29 nm crystallite size and 279.52 crystallites. The peak at 10.15° dominates the XRD pattern of graphene oxide which denotes the (001) plane of GO with an interlayer spacing of 0.87 nm. The d spacing has increased with the transformation of graphite to GO (0.33 to 0.84 nm) due to the insertion of the oxygen groups. The XRD pattern of rGO depicts a peak a at 10.14 corresponding to the (001) plane of GO, indicating the incomplete reduction of GO occurring in the presence of NaBH_4_. Meanwhile the peak at 25.81 corresponds to the (002) plane with an interlayer distance of 0.35 nm (Fig. 1a). The XRD patterns of the composites prepared by coupling TiO_2_ and CuO reveal the peaks corresponding to the crystalline planes of both materials where the composites made of larger amounts of TiO_2_ are dominated with the peaks corresponding to the P25 TiO_2_. The patterns with more CuO are abundant with prominent peaks attributed to CuO (Fig. 1b). In all the composites main peaks belong to the component at lesser proportion are also present. This general trend was observed in the XRD patterns of the composites that were fabricated by coupling with GO and rGO as shown in Fig. 1c and d, respectively.

### Raman analysis

**Figure 2:**
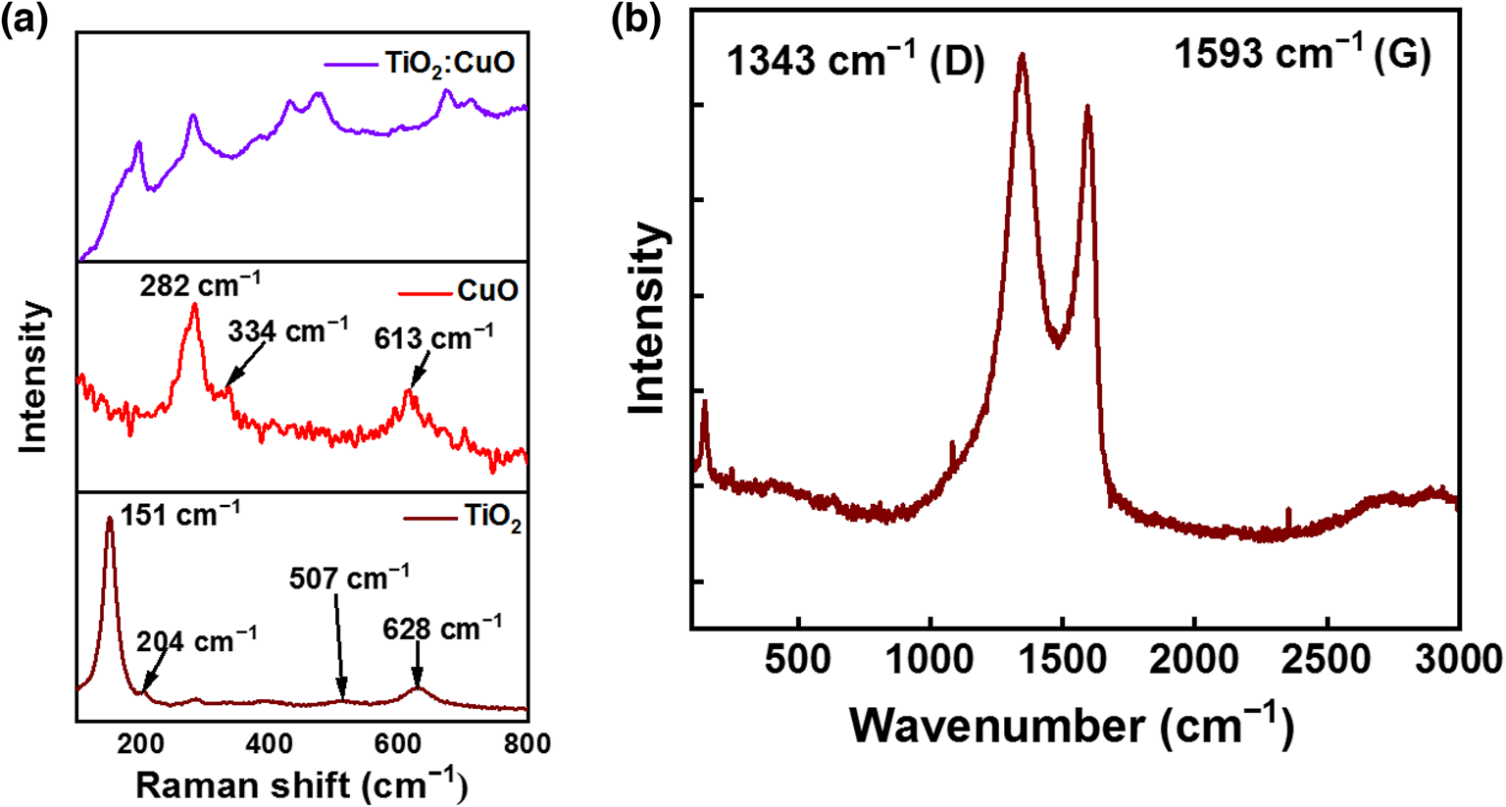
Raman spectra of (**a**) TiO_2_, CuO and GO (**b**) GO.

Raman spectra were collected to analyze the materials’ crystallographic orientation (Fig. 2). The Raman spectrum of pure TiO_2_ shows bands at 151 (E_g_), 204 (E_g_), 397 (B_1g_), 507 (B_1g_) and 628 (E_g_) cm^−1^ and that of pure CuO exhibited bands at 282 (A_g_), 334 (B_g_), and 613(B_g_) cm^−1^ vibrational modes. Main bands of those two are present in TC (1:1) composite (Fig. 2a). The Raman spectrum of GO (Fig. 2b) depicts the D and G band, respectively, at 1343 and 1593 cm^−1^. They correspond to the ring breathing mode of sp^2^ carbon rings and bond stretching of all pairs of the sp^2^ atoms in both rings and chains, respectively^40^.

### Morphological analysis

**Figure 3:**
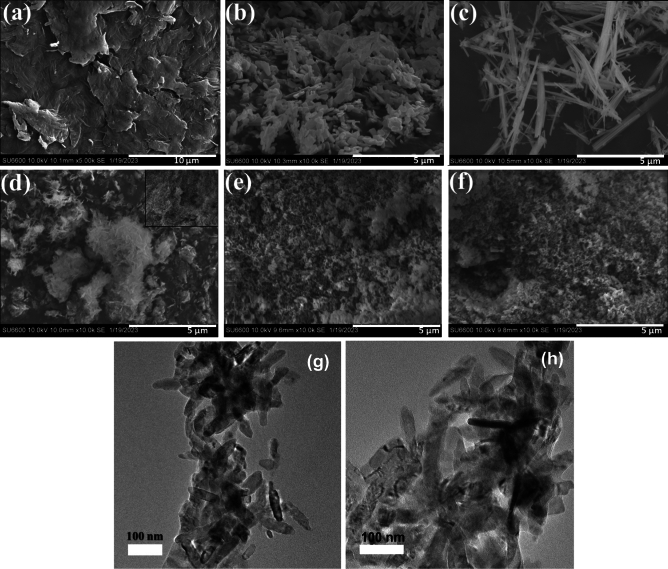
SEM images of (**a**) GO (**b**) CuO (**c**) TiO_2_ (**d**) TC (1:1) (**e**) TCG (1:1) (**f**) TCrG (1:1) (**g**) and (**h**) TEM image of TCG (1:1).

SEM images were generated to study the surface 3D morphology of the synthesized materials (Fig. 3). The image of GO (Fig. 3a) displays the crumpled and wrinkled lamella structure obtained from the oxidation of graphite. The homogeneous graphene sheets are folded and the edges of the new individual GO sheets including the kinked and crumpled structure are very distinguishable. Oxygenated groups existing on the GO produce the wrinkles where the oxidation occurs at the edges and the surface of the graphite flakes. Further, the oxidation extends to the middle of the carbon, adding more oxygenated groups between the sheets which increases the interlayer distance. Moreover, ultrasonication conducted in the presence of the coupled metal oxides further increases the interplanar distance^41^. SEM image of CuO given in Fig. 3b shows new architectures of CuO assembled to small micro rods with some irregular microplates-like structures with distorted edges produced during the hydrothermal treatment. TiO_2_ after being hydrothermally treated in 10 M NaOH appear to be in the form of microrods of with different lengths ranging 0.75–10.5 µm and approximately 180 nm in diameter (Fig. 3c). The TC (1:1) composite appears to be microflakes as shown in the SEM image (Fig. 3d) where the small micro rod-like architecture evident in CuO and long microrods present in pure TiO_2_ have disappeared. They have been replaced by microflakes and the insert of Fig. 3d shows a detailed structure of the plates’ arrangement. However, the same TiO_2_/CuO, once coupled with GO hydrothermally (TCG (1:1)) (Fig. 3e) and then reduced by NaBH_4_ to produce rGO (Fig. 3f), exhibits a random irregular structure where an exact shape cannot be identified. During the second hydrothermal treatment in the presence of GO and then upon exposure to the reducing environment in the presence of NaBH_4_, the proper regular rod-like structure disappeared.

The TEM image of TC (1:1) was acquired to study morphological details at the nanoscale with higher resolution. Small nanorods which were not clearly distinguishable on the SEM image were present, suggesting that the composite consisted of both micro and nanorods. However, the lengthy TiO_2_ microrods present in SEM images were not identifiable either in the TEM image. Hydrothermal treatment is a heterogeneous reaction in which the precursor materials synthesized in vitro in aqueous medium are treated above the ambient temperature and pressure. The mixtures are treated at a temperature higher than the boiling point of water and consequently, the pressure in the hydrothermal vessel is increased above the atmospheric temperature. This synergistic effect of high temperature and pressure provides highly crystalline materials. Further, different morphologies are being created during hydrothermal treatment. This is because during the prolonged exposure of the precursor materials to high temperature and pressure, the initially obtained structures after the nucleation further grow in the direction of one atomic plane and produce rod-like architectures^42,43^.

### XPS analysis

**Figure 4:**
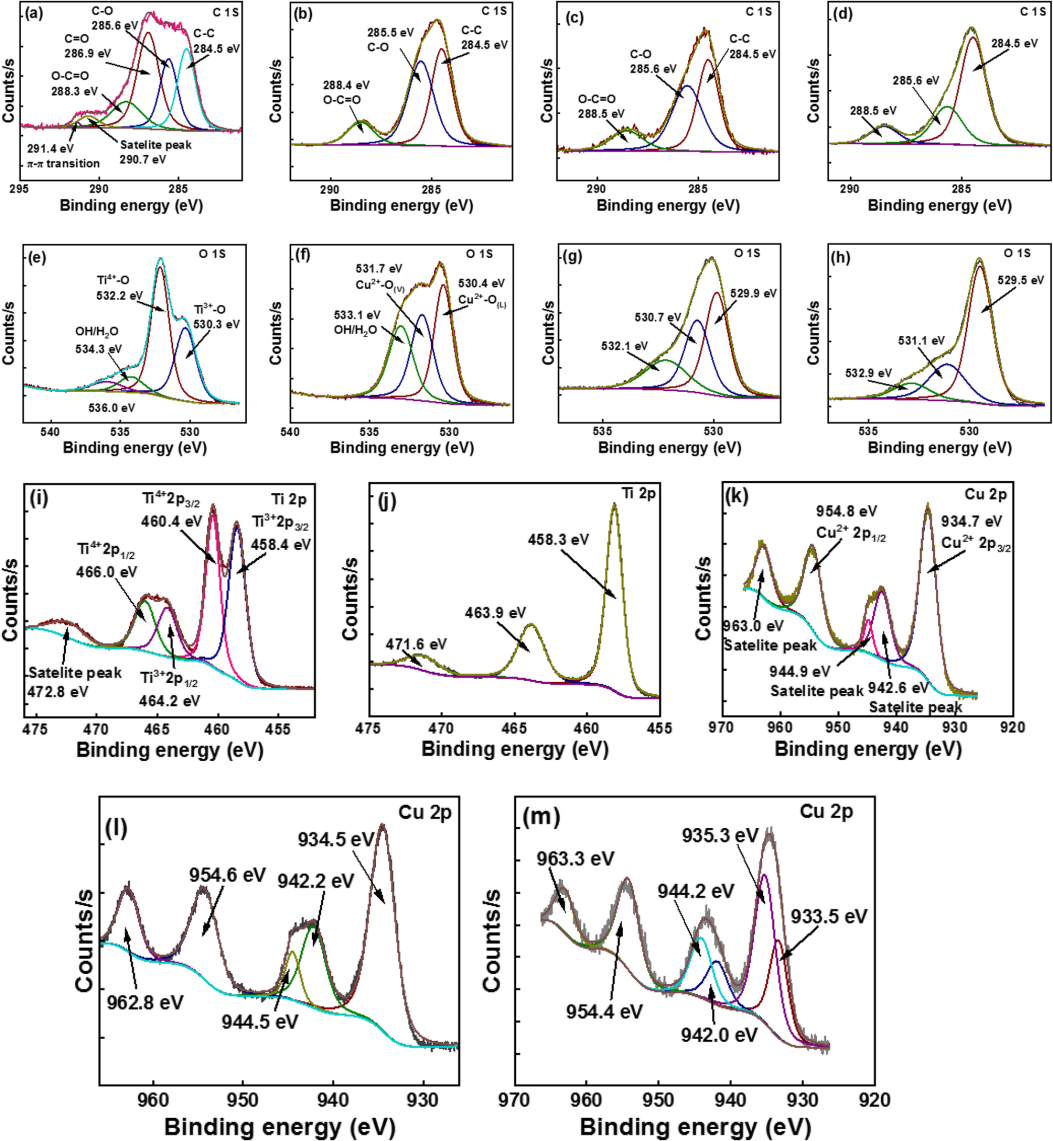
Higher resolution spectra of C 1s (**a**) TiO_2_ (**b**) CuO (**c**) TC (1:1) (**d**) TCG (1:1), O 1s of (**e**) TiO_2_ (**f**) CuO (**g**) TC (1:1) (**h**) TCG (1:1), Ti 2p of (**i**) TiO_2_ and (**j**) TC (1:1), Cu 2p of (**k**) CuO, (**l**) TC (1:1) (**m**) TCG (1:1).

The synthesized materials’ surface was analyzed by XPS where the survey spectra provided the surface elemental composition whereas the higher resolution XPS spectra give a more detailed description of the different chemical environments of each element present. The higher resolution spectrum of C 1s of TiO_2_ (Fig. 4a) is deconvoluted to five peaks at 284.5, 285.6, 286.9, 288.3, 290.7 and 291.4 eV. These are attributed to sp^2^ hybridized graphitic C–C, C–O, C=O, O–C=O, satellite peak and π–π transitions^44^. The C 1s higher resolution spectra of CuO nanomaterials (Fig. 4b) was deconvoluted to three peaks at 284.5, 285.6 and 288.5 eV, which are attributed to C–C, C–O, and O–C=O. Meanwhile similar chemical environments of carbon were observed with TC (1:1) and TCG (1:1) (Fig. 4c and d), respectively^17^. The higher resolution spectrum of O 1s of TiO_2_ (Fig. 4e) was deconvoluted to four peaks at 530.3, 532.2, 534.3 and 536.0 eV and shown here is the presence of Ti^3+^–O, Ti^4+^–O, and OH/H_2_O; the latter can be ascribed to shake-up feature. Indicated here is that both the Ti^3+^ and Ti^4+^ states are present in pure TiO_2_. Three peaks appear in the detailed higher resolution spectrum of O 1s of CuO (Fig. 4f) centered at 530.4, 531.7 and 533.1 eV, which are respectively assigned to Cu^2+^–O_(L)_, Cu^2+^–O_(V)_ and OH/H_2_O. Three different chemical environments of Oxygen–Oxygen bound to metals like Ti and Cu at lower and higher oxidation states and C–O–C are shown by the three peaks centered at 529.9, 530.7 and 532.1 eV in the O 1s higher resolution spectrum of TC (1:1) (Fig. 4g). Similar behavior was observed in TCG (1:1) (Fig. 3h). The higher resolution spectrum of Ti 2p of TiO_2_ shown in the Fig. 4i exhibits five peaks at 458.4, 460.4, 464.2, 466.0 and 472.8 eV which are attributed to 2p_3/2_ of Ti^3+^, 2p_3/2_ of Ti^4+^, 2p_1/2_ of Ti^3+^, 2p_1/2_ of Ti^4+^; the latter can be attributed to the satellite^45,46^. However, the higher resolution spectrum of Ti 2p of TC (1:1) (Fig. 4j) is deconvoluted to three peaks at 458.5, 464.2 and 471.7 eV, which indicates the presence of 2p_3/2_ of Ti^4+^,2p_1/2_ of Ti^4+^ and the latter is ascribed to the shake-up feature. There are six different chemical environments on Cu as shown by the higher resolution spectra of Cu 2p of CuO, TC (1:1) and TCG (1:1) (Fig. 4k, l and m, respectively). The main four peaks are attributed to 2p_3/2_ of Cu^+^, 2p_3/2_ of Cu^2+^, 2p_1/2_ of Cu^+^, and 2p_1/2_ of Cu^2+^ ^47^. The survey spectra shown in Supplementary Fig. 1a, b, c and d display the elemental distribution on the surface of TiO_2_, CuO, TC (1:1) and TCG (1:1), respectively.

### UV–Visible diffuse reflectance spectroscopic analysis

**Figure 5:**
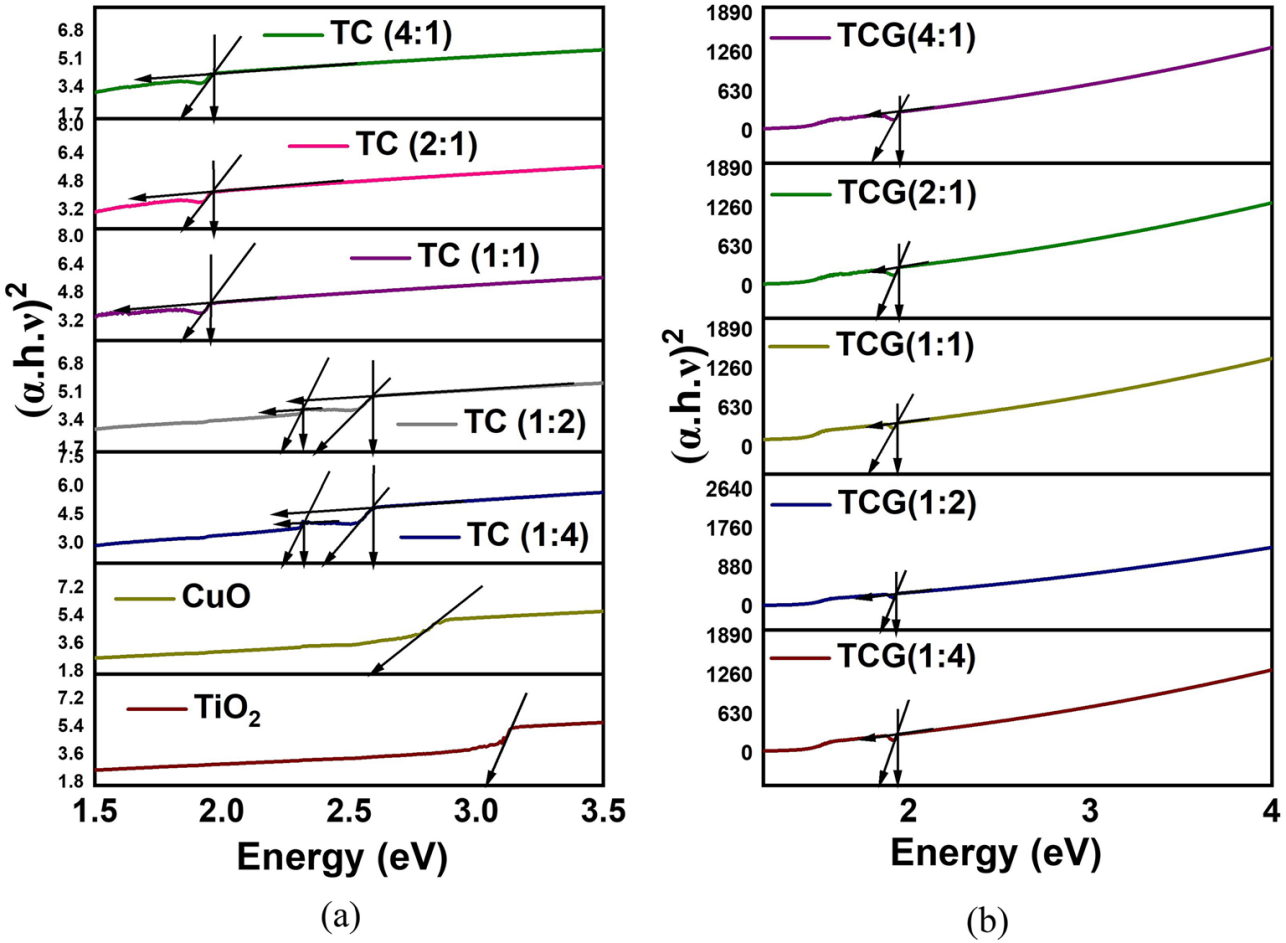
Tauc plots correspond to indirect transitions of (**a**) TC composites (**b**) TCG composites.

The optical absorption behavior of the synthesized nanocomposites was analyzed using UV–Visible spectroscopy. It could be seen that the absorption edge lies at 400 nm for TiO_2_, suggesting the UV sensitive nature of the semiconductor. Meanwhile CuO and the TiO_2_/CuO, TiO_2_/CuO/GO and TiO_2_/CuO/rGO composites show a shift of the absorption edge towards the visible range and this suggests the composites’ improved visible light sensitivity. This resulted due to the coupling of TiO_2_ with CuO and GO/rGO. Tauc plots were constructed using the formula shown below to determine the band gap of the synthesized nanomaterials:

$$ \left( {{\text{ahv}}} \right)^{{\text{n}}} = {\text{ A}}\left( {{\text{hv}} - {\text{Eg}}} \right) $$

where: hv, A, Eg and a represent the photon energy, absorption coefficient, band gap energy and a constant, respectively^48^, n = 2 denotes the direct transitions and n = 1/2 indicates indirect transitions. The plots corresponding to indirect transitions are shown in Fig. 5.

UV–Visible absorption spectra and the plots of direct transitions are shown in Supplementary Figs. 2 and 3. The behavior of the plots suggests that indirect transition is more favorable in the synthesized composites. The band gap of TiO_2_, and CuO are found to be 3.0 and 2.6 eV. The tauc plots of TC (1:4) and TC (1:2) showed a different behaviour where two vertical portions corresponding to two band gaps were identified at 2.6 and 2.3 eV. In both composites Cu is present in higher proportion compared to TiO_2_ and hence, it is evident that CuO and Cu doped to TiO_2_ are present in the composite. In the other composites where Ti and Cu are mixed equally (TC (1:1)) and Ti present in higher proportions (TC (4:1) and TC (1:4)) only one sudden rise of absorption corresponding to electron excitement from the valence band to conduction band could be seen corresponding to a band gap of 1.95 eV indicating the presence of only Cu doped TiO_2_. This behaviour is further discussed in the section which describes the mechanism. The band gap of a photocatalyst is influenced by the size and shape of the nanomaterial in addition to the semiconductor’s band alignment. Particle size and band gap are typically inversely correlated. The band gap, where the band gap values grow as the particle size decreases, is significantly influenced by the size of the nanoparticles. As the size of the particle diminishes, electrons in the conduction band and holes in the valence band become more restricted, increasing the band gap between the valence and conduction bands. The band gap values are influenced by the nanomaterials’ form. Changes in the size and form of the nanomaterials affect the volume-to-surface area ratio, which in turn affects the number of surface atoms and, ultimately, the cohesive energy. Consequently, the change in size and shape causes the band gap to vary at the nanoscale^49^. Hence, a combination of circumstances led to the obtained band gap values. The linked heterojunction composites’ apparent photocatalytic activity is clear.

### Photocatalysis

**Figure 6:**
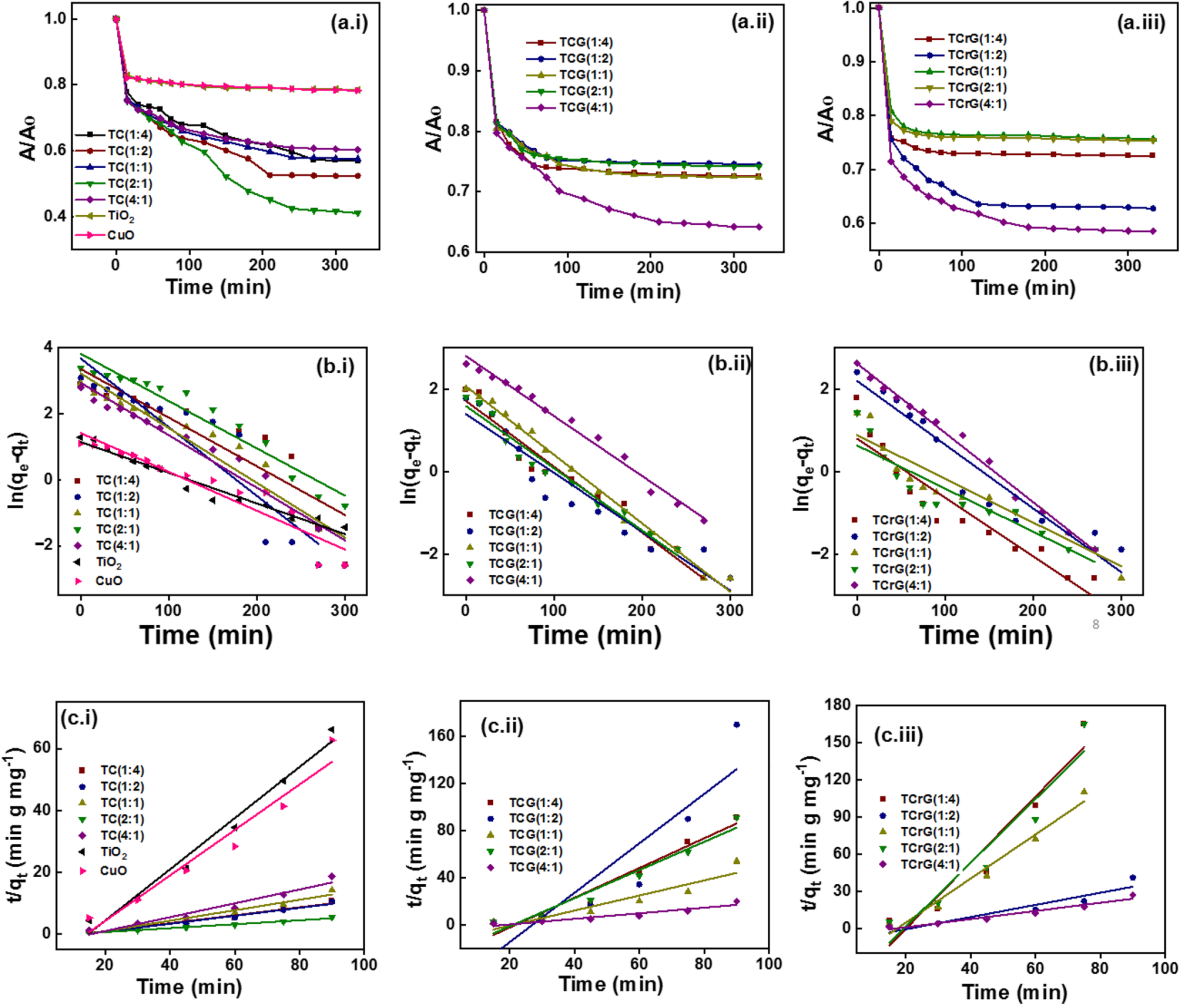
(A/A_0_) versus time of (a_i_) TC (a_ii_) TCG (a_iii_) TCrG composites, First order kinetics (b_i_) TC (b_ii_) TCG (b_iii_) TCrG composites, and second order kinetics (c_i_) TC (c_ii_) TCG (c_iii_) TCrG.

Adsorption kinetics of MB adsorbing to the synthesized nanocomposites was studied by shaking the nanomaterials in 50 mg/ml (Fig. 6). The variations of A/A_0_ with time of TC, TCG and TCrG composites are shown in Fig. 6ai, a_ii_, a_iii_, first order kinetics of TC, TCG and TCrG in Fig. 6bi, b_ii_, b_iii_, second order kinetics in Fig. 6ci, c_ii_, c_iii_. The time taken to reach the adsorption desorption equilibrium was monitored. In general, all the composites reached the adsorption desorption equilibrium in 270 min maximum. The kinetics of mass transfer in the adsorption of methylene blue to the adsorbents is mainly governed by external diffusion. This involves: firstly, the mass transfer of the methylene blue molecules to the external surface of the adsorbent; secondly, internal diffusion which involves the diffusion of the methylene blue molecules through the pores of the adsorbents; and thirdly, adsorption of the methylene blue molecules to the adsorbents via physisorption or chemisorption. As shown in Table 1 according to the coefficient of determination (R^2^) the adsorption of MB follows the second order kinetics which suggests chemisorption of MB to the nanocomposites.

**Table 1:** The coefficient of determination (R^2^) and the rate constant values for first order and second order kinetics of adsorption.

Composite	1st order kinetics		2nd order kinetics	
	K (min^−1^)	R^2^	K (mg g^−1^ min^−1^)	R^2^
TC (1:4)	1.4 × 10^−2^	0.8923	1.28 × 10^−1^	0.9528
TC (1:2)	2.1 × 10^−2^	0.8501	1.23 × 10^–1^	0.9684
TC (1:1)	1.6 × 10^–2^	0.9335	1.69 × 10^–1^	0.9743
TC (2:1)	1.4 × 10^–2^	0.9484	6.12 × 10^–1^	0.9784
TC (4:1)	9.3 × 10^–3^	0.9621	2.24 × 10^–1^	0.9747
TiO_2_	1.1 × 10^–2^	0.8915	8.28 × 10^–2^	0.9899
CuO	1.6 × 10^–2^	0.9547	4.23 × 10^–2^	0.9742
TCG (1:4)	1.4 × 10^–2^	0.9109	7.23 × 10^–1^	0.9899
TCG (1:2)	1.6 × 10^–2^	0.9215	9.14 × 10^–1^	0.9742
TCG (1:1)	1.5 × 10^–2^	0.9627	2.57 × 10^–1^	0.9881
TCG (2:1)	1.4 × 10^–2^	0.9858	7.26 × 10^–1^	0.9923
TCG (4:1)	1.4 × 10^–2^	0.8703	9.90 × 10^–1^	0.9427
TCrG (1:4)	1.5 × 10^–2^	0.9210	5.50 × 10^–1^	0.9885
TCrG (1:2)	1.0 × 10^–2^	0.8740	2.48 × 10^–1^	0.9017
TCrG (1:1)	1.1 × 10^–2^	0.9210	3.97 × 10^–1^	0.9585
TCrG (2:1)	1.6 × 10^–2^	0.8740	7.7 × 10^–1^	0.9620
TCrG (4:1)	1.2 × 10^–2^	0.9818	6.84 × 10^–1^	0.9897

MB molecules are positively charged whereas the catalyst surface is negatively charged, which facilitates the electrostatic interactions between the dye molecules and the substrate surface. The MB molecules form π–π interactions with the aromatic rings in GO and moreover, the dye molecules form electrostatic interactions with the negatively charged oxygen-rich functional groups generated on the carbon surface during the formation of GO via oxidation. The presence of oxygen-rich functional groups was further discussed in the XPS analysis. The C/O ratio in rGO is much higher due to the reduction of the oxygen functionalities and hence limits the MB being adsorbed via electrostatic bonds. Nonetheless, adsorption through π–π interactions still occurs.

**Figure 7:**
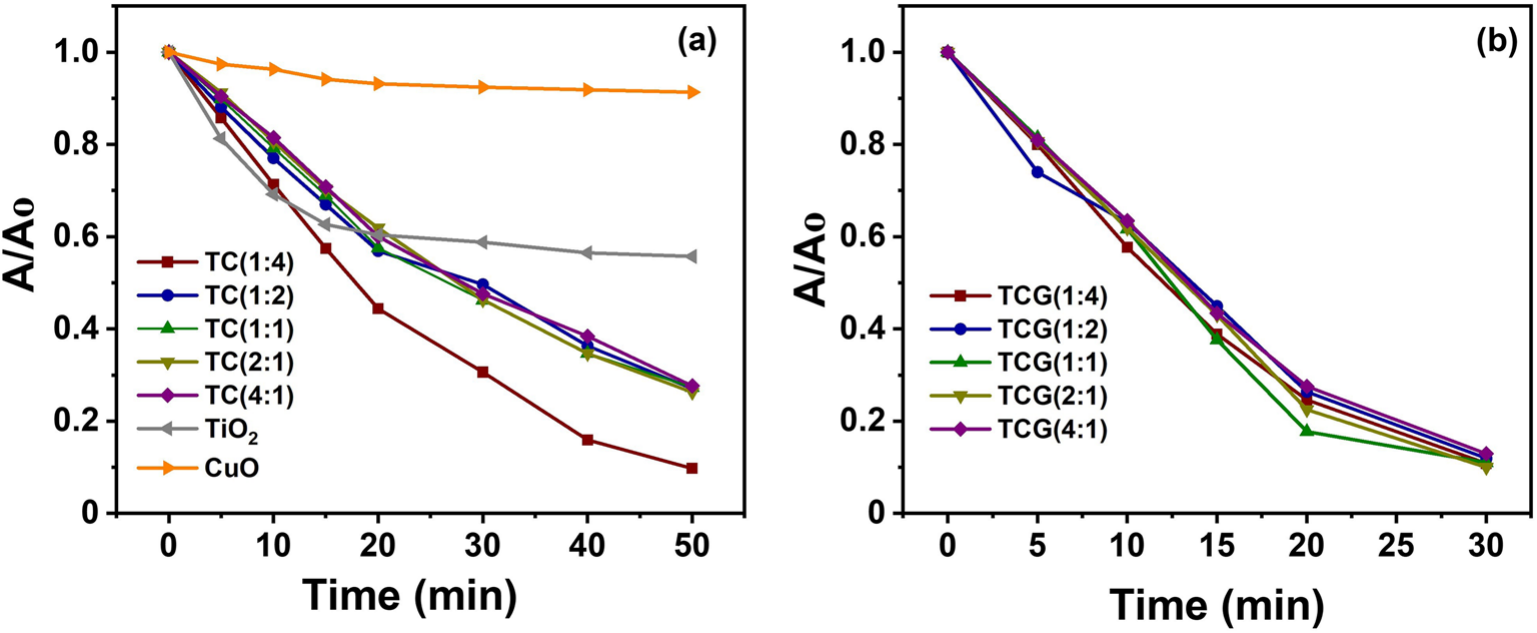
Variation of A/A_0_ with time in photodegradation of MB in the presence of TC and TCG.

The variations of the A/A_0_ of photodegrading MB in the presence of TC and TCG samples are shown in Fig. 7. In general, it indicates that there is a slight reduction in the absorbance of MB in the presence of CuO while the highest reduction in absorbance was obtained in the presence of TC (1:4) (Fig. 7a). A/A_0_ of MB in the presence of all the TCG catalysts reached almost 0.1 within 30 min as shown in Fig. 7b. Photocatalysis in the presence of the catalysts are described in detail with the first order kinetic plots.

The photocatalytic activity of the synthesized composites was determined based on the degradation of methylene blue under visible light irradiation in the presence of persulfate ions. This is known to enhance the photocatalytic activity due to the production of the SO_4_^−·^ and OH^·^, made possible by the reduction of S_2_O_8_^2−^ as shown in the reactions shown below:

$$ {\text{S}}_{{2}} {\text{O}}_{{8}}^{{{2} - }} + {\text{ e}}^{ - } \to {\text{SO}}_{{4}}^{ - \cdot } + {\text{ SO}}_{{4}}^{{{2} - }} $$

$$ {\text{SO}}_{{4}}^{ - \cdot } + {\text{ OH}}^{ - } \to {\text{ SO}}_{{4}}^{{{2} - }} + {\text{OH}}^{ \cdot } $$

$$ {\text{S}}_{{2}} {\text{O}}_{{8}}^{{{2} - }} + {\text{h}}\nu \to {\text{2SO}}_{{4}}^{ - \cdot } $$

$$ {\text{S}}_{{2}} {\text{O}}_{{8}}^{{{2} - }} + {\text{O}}_{{2}}^{ - \cdot } \to {\text{SO}}_{{4}}^{{{2} - }} + {\text{SO}}_{{4}}^{ - \cdot } + {\text{O}}_{{2}} $$

**Figure 8:**
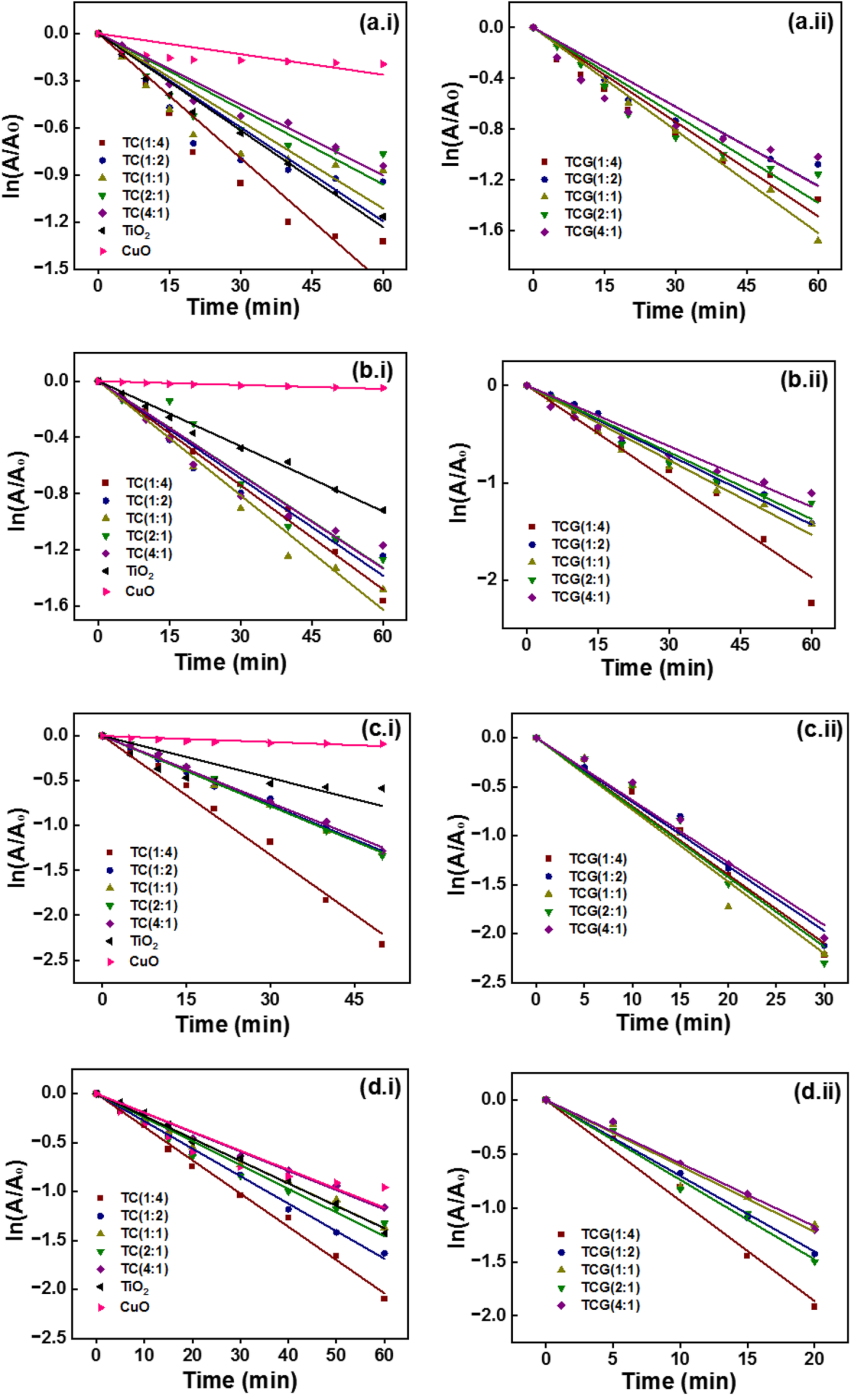
The first order kinetic plots of TC and TCG composites at different persulfate concentrations: 1 mM (Fig. 7 (a1) and (a2)), 2 mM (Fig. 7 (b1) and (b2)), 4 mM (Fig. 7 (c1) and (c2)) and 8 mM (Fig. 7 (d1) and (d2)).

The rate constant for the photodegradation of MB in the presence of Pure TiO_2_ (P25) as the photocatalyst was higher than that when CuO was present at all the concentrations of the persulfate used. The photocatalytic activities of the TC composites at different ratios were determined at different persulfate concentrations, namely 1, 2, 4 and 8 mM. It was observed that the photocatalytic activity of the composites fabricated at the different compositions behaved differently. The first order kinetic plots of TC and TCG composites at different concentrations are shown in Fig. 8 as 1 mM (Fig. 8a1 and a2), 2 mM (Fig. 8b1 and b2), 4 mM (Fig. 8c1 and c2) and 8 mM (Fig. 8d1 and d2). The rate constant for the photodegradation of MB increased with the rising concentration of persulfate ions to 4 mM, and then diminished with a further increase in the concentration to 8 mM in the presence of TC (4:1). It is evident that the photocatalytic activity increased with increasing concentration of persulfate up to the desired value. Once the concentration exceeds such a value, part of persulfate will participate in the generation of free radicals. The excess will retard the contact time possibilities between MB and free radicals and subsequently generate less photocatalytic activity^50^. Further, at higher concentrations of persulfate ions sulfate and hydroxyl radicals tend to quench as shown in the following equations:

$$ {\text{S}}_{{2}} {\text{O}}_{{8}}^{{{2} - }} + {\text{SO}}_{{4}}^{ - \cdot } \to {\text{ SO}}_{{4}}^{{{2} - }} + {\text{ S}}_{{2}} {\text{O}}_{{8}}^{ - \cdot } $$

$$ {\text{S}}_{{2}} {\text{O}}_{{8}}^{{{2} - }} + {\text{OH}}^{ \cdot } \to {\text{ OH}}^{ - } + {\text{ S}}_{{2}} {\text{O}}_{{8}}^{ - \cdot } $$

Further, at larger concentrations the reaction between radicals given below become more significant, reducing the availability of the free radicals and hence any photocatalytic activity^51^:

$$ {\text{SO}}_{{4}}^{ - \cdot } + {\text{ SO}}_{{4}}^{ - \cdot } \to {\text{ 2SO}}_{{4}}^{{{2} - }} $$

$$ {\text{OH}}^{ \cdot } + {\text{ OH}}^{ \cdot } \to {\text{ H}}_{{2}} {\text{O}}_{{2}} $$

$$ {\text{SO}}_{{4}}^{ - \cdot } + {\text{ OH}}^{ \cdot } \to {\text{ HSO}}_{{4}}^{ - } + {\text{ 1/2 O}}_{{2}} $$

Similar behavior was observed in the presence of TC (2:1) and TC (1:1). The rate constants of photodegrading MB in the presence of TC (4:1), TC (2:1) and TC (1:1) increased by 1.65, 1.62 and 1.4 times when the concentration increased from 1 to 4 mM. The rate constant for the photodegradation of MB increased when the concentration of persulfate also increased to 8 mM in the presence of TC (1:2). This is 1.5 times higher than that resulted with 1 mM but such a trend was not observed with TC (1:4). The behavior of the GO-coupled TC composites was different from that of the uncoupled TC composites. The rate constantly rose with increasing persulfate concentration to 4 mM in both TCG (4:1) and TCG (1:1) by 3.15 and 2.9 times, respectively, and then decreased with further increment to 8 mM.

The photocatalytic activity increased with increasing concentration with TCG (2:1) and TCG (1:2) by 3.3 and 3.5 times at 8 mM compared to those obtained when 1 mM of persulfate in the whole concentration range tested. Like the uncoupled TC (1:4) no trend in the photocatalytic activity was observed in the TCG (1:4). The photocatalytic activity of the TC composites in general has increased with coupling with GO in TCG composites at low persulfate concentrations (1 and 2 mM). It increased 2–3 times when 4 and 8 mM persulfate were used. Suggested here is that the photocatalytic activity of TC was enhanced by coupling with GO due to the enhanced separation of the electron hole pairs and this facilitated charge transfer along the GO sheets due to their high conductivity.

#### Weight of the photocatalyst

**Figure 9:**
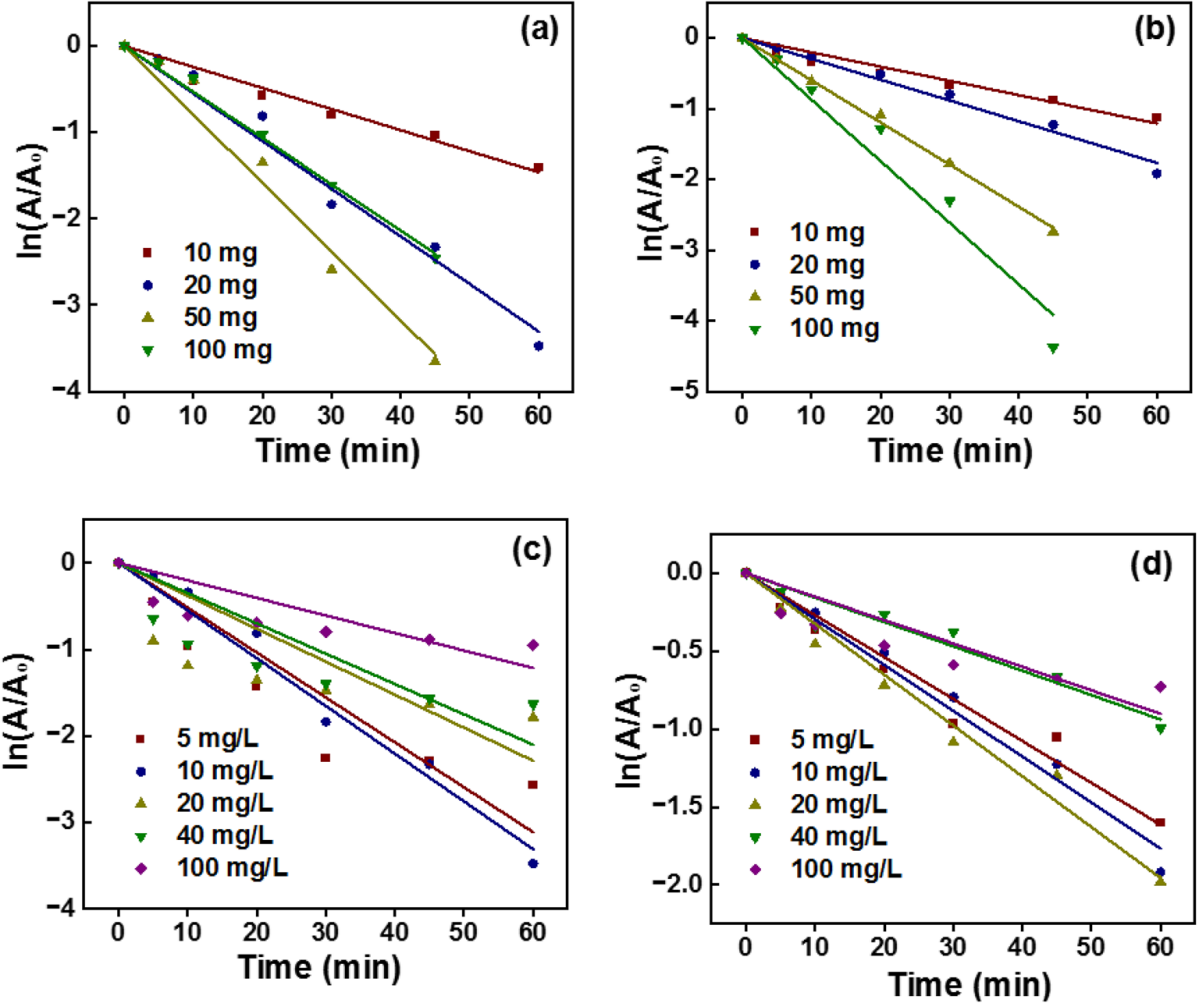
Variations in the weight of the catalyst of (**a**) TC and (**b**) TCG and the concentration of the dye (**c**) TC and (**d**).

The weight of the TC and TCG catalysts was varied to study the impact of the catalyst dosage on photocatalytic activity (Fig. 9a and b, respectively). It was observed that the rate of photodegrading MB increased from 2.4 × 10^–2^ min^−1^ to 7.9 × 10^–2^ min^−1^ which is a 3.2-fold increment when the dosage of the catalyst rose from 10 to 50 mg. However, it was reported that with further increase in the weight of the catalyst to 100 mg the rate dropped to 5.3 × 10^–3^ min^−1^. This was lower than what was obtained when 10 mg of the catalyst was used. The number of active sites increases when the weight of catalyst also increases, which makes possible the MB’s adsorption to the surface of the catalyst. Further, the charge carriers produced by the catalysts increase with the increase of the catalyst weight, subsequently leading to the production of higher concentrations of radicals which degrade the MB molecules. However, when the weight of the catalyst is further increased the rate of degradation falls dramatically.

The catalyst particles collide with each other and mask the active sites of other particles, thus limiting the available active sites exposed to the reactant MB molecules. Furthermore, the reach of the photons produced from the visible light source is limited due to the same effect reducing the production of the radicals eventually diminishing the oxidation of MB. Moreover, O_2_ is created as a product as shown in reaction number. It traps the surface of the catalyst and hence MB molecules reaching the surface of the catalyst would be limited, decreasing the total degraded MB. Interestingly, the catalysts coupled with GO showed a different behavior where the rate constant of photodegrading MB increased as the catalyst increased in weight. The rate constant resulting with 10 mg of the coupled composite (2 × 10^–2^ min^−1^) increased 4.35 times to 8.7 × 10^–2^ min^−1^ with the use of 100 mg of the catalyst. This is due to the presence of GO which serves as a matrix in which the catalyst particles are dispersed.

Hence, with an increase in the catalyst dosage more active sites with more oxygen-related functional groups are available on the GO surface to be exposed to the MB molecules, thereby enhancing the adsorption process. Since the catalyst particles are well dispersed on the GO matrix, radicals produced easily interact with the well adsorbed MB molecules and facilitate the oxidation. As more charge carriers are produced with increasing concentration of the catalysts, they further enhance the degradation of MB. Masking of the catalyst surface is not expected as the GO would result in improved adsorption of the MB molecules which is a key in producing higher photocatalytic activity. All the R^2^ values and the rate constants at different catalyst dosages are summarized in Table 2.

**Table 2:** R^2^ values and the rate constants at different catalyst dosages.

Weight (mg)	TC (1:4)		TCG (1:4)	
	R^2^	K (min^−1^)	R^2^	K (min^−1^)
10	0.9867	2.4 × 10^–2^	0.9794	2.0 × 10^–2^
20	0.9876	5.5 × 10^–2^	0.9924	2.9 × 10^–2^
50	0.9972	7.9 × 10^–2^	0.9985	5.9 × 10^–2^
100	0.9954	5.3 × 10^–2^	0.9753	8.7 × 10^–2^

#### Concentration of the dye

The effect of concentration of MB on the photocatalytic activity of both the TC and TCG catalysts was determined by varying the concentration of MB in the 5–100 mg/ml range (Fig. 9 c and d, respectively). The photocatalytic activity was increased when the concentration of MB rose from 5 to 10 mg/ml as revealed by the increase in the rate constant in the uncoupled photocatalysts. Meanwhile the rate of the reaction increased with the increase of the MB concentration from 5 to 15 mg/ml as shown in Table 3. The number of molecules increases with increasing concentration of MB, so more molecules adsorb on the catalyst surface and more molecules tend to capture the radicals. Eventually they are photodegraded and this increases the rate of the reaction. However, the photocatalytic activity decreased with a further increase in the concentration of MB from 10 to 20 mg/ml in the presence of uncoupled and coupled catalysts, respectively.

**Table 3:** R^2^ values and the rate constants at different dye concentrations.

Concentration (mg/L)	TC (1:4)		TCG (1:4)	
	R^2^	K (min^−1^)	R^2^	K (min^−1^)
5	0.9341	5 × 10^–2^	0.9837	2.6 × 10^–2^
10	0.9886	5.5 × 10^–2^	0.9907	2.9 × 10^–2^
20	0.9721	3.8 × 10^–2^	0.9897	3.2 × 10^–2^
40	0.9976	3.5 × 10^–2^	0.9667	1.5 × 10^–2^
100	0.9642	2 × 10^–2^	0.9755	1.5 × 10^–2^

Though the number of molecules increases with a further increase in the concentration of MB the available active sites on a given weight of the catalyst are limited. A higher number of molecules compete for the same number of active sites though the concentration of MB was increased. MB is a bulky molecule and hence although the number of molecules increases in tandem with the increase of the concentration, there are still vacant active sites. Available MB molecules cannot reach the active sites of the catalyst due to the steric hindrance leading to lower photodegradation of MB. The decrease in the rate constant and hence photocatalytic activity was observed as starting from 20 to 10 mg/ml in the presence of coupled and coupled photocatalysts, respectively. This is due to the presence of GO in the coupled photocatalysts which possesses many active sites with which the MB could easily interact compared to the uncoupled photocatalysts. However, at a given concentration the rate constant is lower in the GO coupled photocatalysts compared to the uncoupled photocatalysts. This is because in a given weight the weight of the catalyst in the GO coupled photocatalysts is lower than that of the uncoupled photocatalysts. Subsequently, the concentration of the radicals produced would be smaller and lead to less photocatalytic activity.

**Figure 10:**
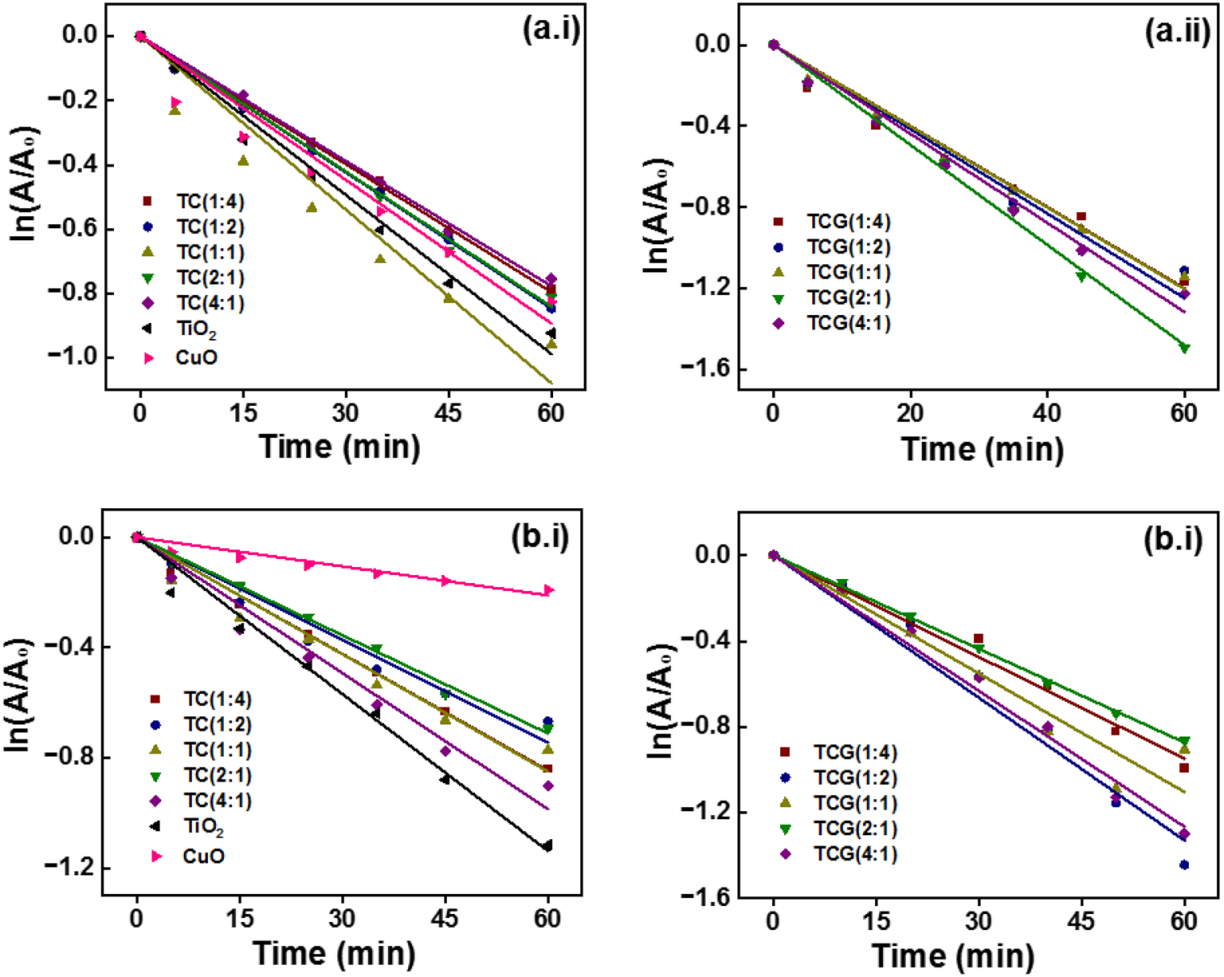
First order kinetics of photocatalysis of (a.i) TC and (a.ii) TCG in the presence of EDTA and of (b.i) TC and (b.ii) TCG in the presence of IPA.

The analysis was carried out in the presence of EDTA and IPA as they scavenge the holes and OH·, respectively, to determine the reactive species. In the presence of EDTA and IPA, the rate constant for the photodegradation of MB dropped in the presence of all the photocatalysts as tabulated in Table 4. The drop in the rate constant in the presence of TiO_2_ was negligible whereas a small decrease was observed in the presence of CuO. Among the TiO_2_/CuO composites, TC (1:4) revealed the highest photocatalytic activity in the presence of persulfate ions (4.4 × 10^–2^ min^−1^) which decreased to 1.3 × 10^–2^ min^−1^ and 1.4 × 10^–2^ min^−1^ in the presence of EDTA and IPA. Among the GO coupled composites, TCG (1:1) demonstrated the most photocatalytic activity (7.35 × 10^–2^ min^−1^) but it reduced to 2.0 × 10^–2^ min^−1^ and 1.8 × 10^–2^ min^−1^, in the presence of EDTA and IPA, respectively, specifically 3.7- and 4.0-fold reductions in photocatalytic activity. Comparatively the drop in the rate of the reaction was higher in GO-coupled photocatalysts (Fig. 10). These outcomes suggest that holes and OH· both contributed to the photodegradation of MB.

**Table 4:** The rate constants of photocatalysis in the presence of EDTA and IPA.

Composite	EDTA		IPA	
	K (min^−1^)	R^2^	K (min^−1^)	R^2^
TC (1:4)	1.3 × 10^–2^	0.9991	1.4 × 10^–3^	0.9971
TC (1:2)	1.4 × 10^–2^	0.9989	1.6 × 10^–3^	0.9866
TC (1:1)	1.7 × 10^–2^	0.9724	1.3 × 10^–3^	0.9857
TC (2:1)	1.4 × 10^–2^	0.9978	1.5 × 10^–3^	0.9976
TC (4:1)	1.2 × 10^–2^	0.9978	1.2 × 10^–3^	0.9894
TiO_2_	1.6 × 10^–2^	0.99339	1.8 × 10^–3^	0.9946
CuO	1.4 × 10^–2^	0.9785	2.2 × 10^–3^	0.9761
TCG (1:4)	1.9 × 10^–2^	0.9882	2.6 × 10^–3^	0.9946
TCG (1:2)	2.0 × 10^–2^	0.9859	2.5 × 10^–3^	0.9885
TCG (1:1)	2.0 × 10^–2^	0.9945	2.8 × 10^–3^	0.9767
TCG (2:1)	2.4 × 10^–2^	0.9977	12.1 × 10^–3^	0.9995
TCG (4:1)	2.2 × 10^–2^	0.9934	2.910^–3^	0.9951

### Reusability

**Figure 11:**
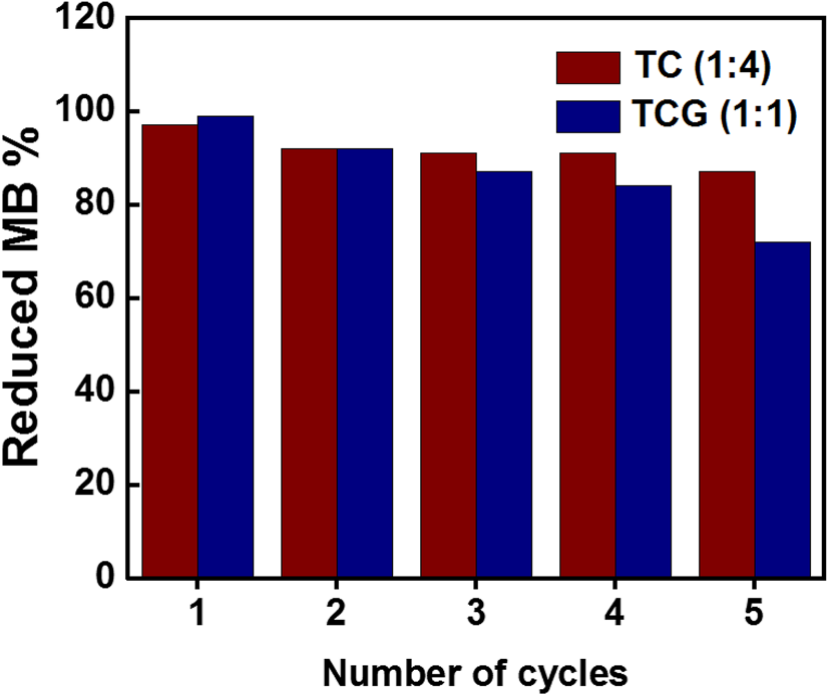
Reusability study of TC and TCG catalysts.

The commercial applicability of the synthesized GO coupled and uncoupled catalysts were tested using the same catalysts for five catalytic reaction runs. What was noticeable was that the photocatalytic activity did not significantly drop in the uncoupled catalyst where the conversion of the photodegradation reduced from 99 to 89% at the fifth run. Conversely, the photocatalytic activity was reduced from 99 to 72% considerably at the fifth run when the GO coupled photocatalyst is used (Fig. 11). In addition to the slight loss of the photocatalysts expected at each run, aggregation of the catalyst particles led to lower surface area and pore volumes reducing the adsorption of MB and hence reducing the photocatalysis. The pores and pore channels are occupied by the pre-adsorbed MB molecules originating from the previous runs moving from the first to the fifth cycle. Additionally, the chemisorbed MB molecules on the active sites are not removed from a simple wash and since the active sites are occupied and the number of vacant sites available for the MB to adsorb decreases, moving to the fifth run the photocatalytic activity also decreases. Further, leaching out of the TiO_2_ and CuO photocatalytic particles from the GO matrix may also have contributed to the considerable loss of photocatalytic activity of the GO-coupled photocatalysts compared to the uncoupled catalysts.

### Mechanism

**Figure 12:**
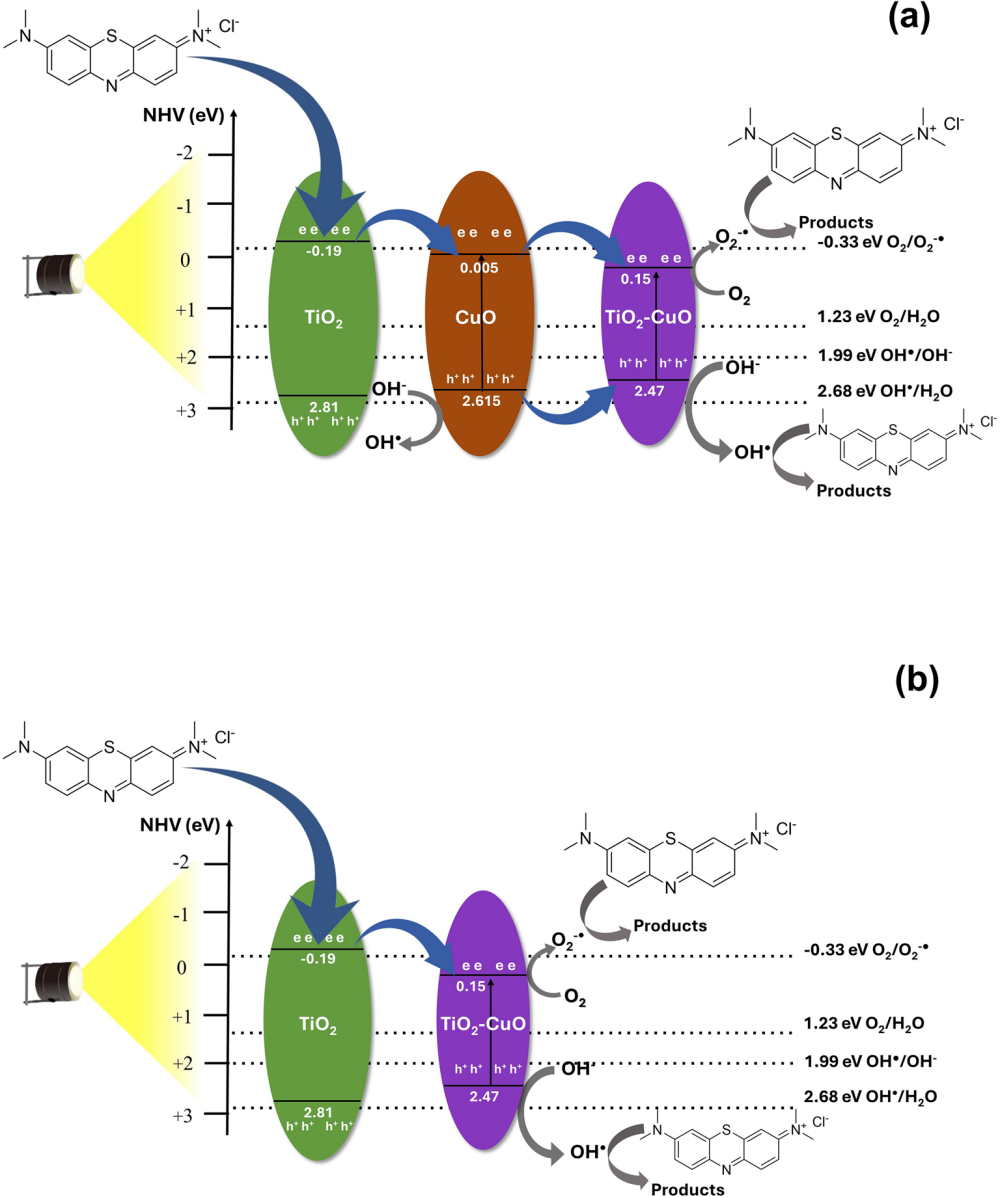
Band alignment of (**a**) TC (1:4) and TC (1:2) and (**b**) TC (1:1), TC (2:1) and TC (4:1) indicating the mechanism of photodegradation.

The heterostructure formed in the photocatalysts is important for determining the mechanism of the photodegradation of MB (Fig. 12).

The band gap values of TiO_2_, CuO and Cu-TiO_2_ determined by the Tauc plots are 3.0, 2.61 and 2.32 eV, respectively. The band edge potentials of the conduction band (E_CB_) and the valence band (E_VB_) of the above semiconductors were calculated by the following formulae, respectively:

$$ E_{CB} = X{-}E^{C} {-}0.5 \, E_{g} $$

$$ E_{VB} = X{-}E^{C} + 0.5 \, E_{g} $$

where: *X* stands for the electronegativity of the semiconductor, which is the geometric mean of the electronegativity of the constituent atoms; and *E*^*C*^ denotes the energy of the free electrons on the hydrogen scale which is approximately 4.5. The X value of both TiO_2_, CuO and Cu-TiO_2_ is 5.81. E_CB_ calculated for TiO_2_ (Rutile), TiO_2_ (Anatase), CuO and Cu-TiO_2_ are − 0.19, − 0.39, 0.005 and 0.15 eV/normal hydrogen electrode (NHE), respectively. Meanwhile the E_VB_ calculated are 2.81, 2.81, 2.61 and 2.47 eV/normal hydrogen electrode (NHE), respectively.

A homojunction is established between Rutile and Anatase of which the VB of both semiconductors at 2.81 eV while the CB of Rutile at a potential − 0.19 eV/normal hydrogen electrode (NHE) is located below the CB of TiO_2_ Anatase (0.39 eV/normal hydrogen electrode (NHE)). However, as a visible light source used for the excitation, neither Rutile nor Anatase would expect to be excited since their band gaps are 3.0 and 3.2 eV, respectively. Heterostructure consisting of TiO_2_, CuO and Cu-TiO_2_ is present in the TC (1:4) which showed the highest photocatalytic activity. The arrangement of the VB and the CB as shown in Fig. 12a suggests that type II band alignment is present in the heterostructure.

Type II heterojunctions are not favorable in photocatalysis because they promote the electron hole pair recombination. Nonetheless, as only a visible light source was used TiO_2_ based semiconductors would not be excited since they are only UV sensitive. Hence, visible light sensitive CuO and Cu-TiO_2_ would only be active once exposed to visible light generated from the LED source used. MB molecules can absorb visible light to generate electrons which transfer from the LUMO level of MB to the CB of TiO_2_. These electrons continue to transfer from the CB of TiO_2_ to the CB of CuO and then to the CB of Cu doped TiO_2_. Meanwhile as CuO and Cu doped TiO_2_ is visible-sensitive it also gets excited once it is exposed to visible light and excites electrons to the CB, leaving holes at the VB. Then the electrons at the CB of CuO move to the CB of the Cu -TiO_2_ and thus create a high electron concentration at the CB of Cu-TiO_2_. Holes tend to migrate from the VB of CuO to the VB of Cu-TiO_2_ increasing the concentration of holes. As the bands exhibit a type II band alignment, high electron concentration accumulated at the CB of Cu-TiO_2_ tends to recombine with the holes at the VB. However, in the composites TC (1:1) and others with increasing Ti proportion (TC(2:1) and TC (4:1) all only TiO_2_ and Cu-TiO_2_ present where the electrons move to the CB of TiO_2_ from the LUMO level of MB molecules migrate to the CB of Cu-TiO_2_ while Cu-TiO_2_ itself excite electrons from the VB to CB leaving holes at the VB (Fig. 12b). Therefore, it can be considered that the concentration of the radicals generated at the heterojunction with TC (1:4) and TC (1:2) are higher than those produced at the heterojunctions produced in TC (1:1), TC (2:1) and TC (4:1). Conversely, the photocatalysis was negligible in the absence of persulfate ions, indicating that the active groups originated mainly from the externally added persulfate ions rather than the photogenerated charge carriers. However, the charge carriers are essential for the generation of the radicals from the persulfate ions added externally. Therefore, although a small concentration of charge carriers is produced, they are very important in generating reactive radicals such as SO_4_^−·^and OH^·^. The conductive nature of GO facilitates the charge separation and thus enhances the photocatalysis.

#### Effect of reduced graphene oxide

**Figure 13:**
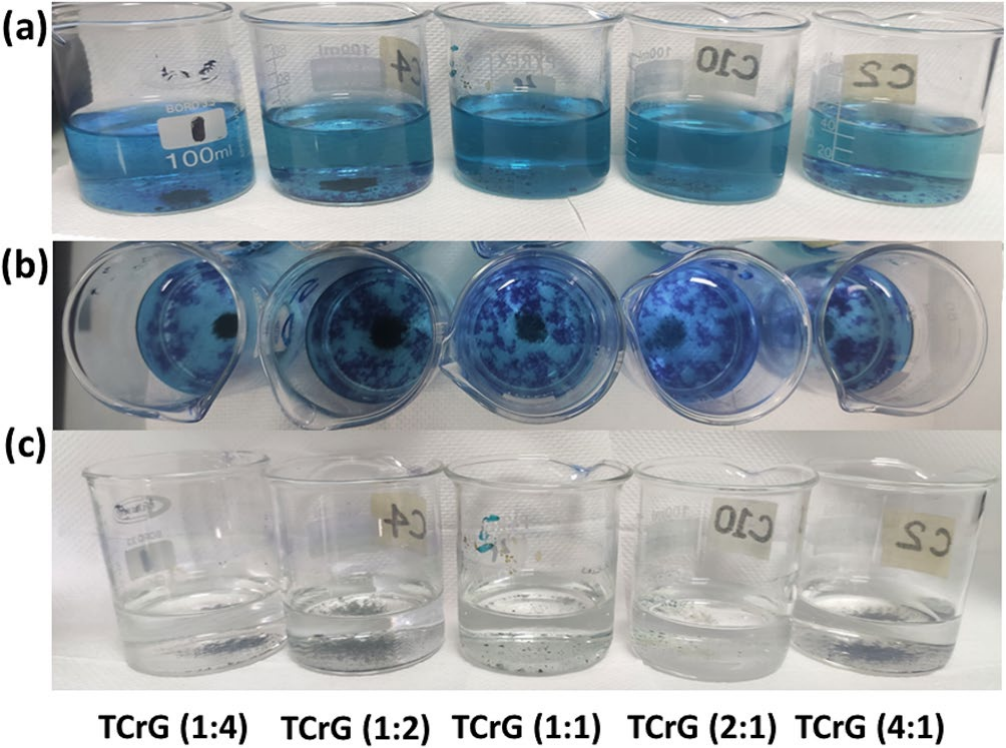
Effect of TCrG on coagulation of MB (**a**) right after the addition of the catalysts (**b**) after one hour (**c**) after 24 h of adding the catalysts.

In the presence of TiO_2_/CuO/Reduced graphene oxide and persulfate ions MB tend to coagulate and with time they were settled down producing a transparent solution as shown in Fig. 13. Coagulation-flocculation phenomenon is governed by charge neutralization. MB is a cationic dye and TCrG whose surface is negatively charged neutralizes the MB molecules. Upon reduction of graphene oxide, the oxygen-related functional groups are reduced and hence compared to graphene oxide a well-developed π electron system is present. Particularly, in reduced graphene oxide the electron flow is higher because the conductivity of reduced graphene oxide is higher compared to graphene oxide which further neutralizes the positively charged MB molecules. Consequently, the MB molecules tend to agglomerate.

In the presence of persulfate ions such produced agglomerates were brought together as the negatively charged persulfate ions further neutralize cationic MB molecules serving as a flocculant; this results in the MB agglomerates settling and leaving a clear solution. The experiment was conducted in both light and dark to find out if any photocatalysis would occur in addition to the coagulation-flocculation. It was observed that a clear solution with the catalyst settling at the bottom resulted in the samples being exposed to light while blue color clumps were settled at the bottom or floated to the surface in the samples exposed to dark. This suggests that despite the coagulation-flocculation process being faster in the presence of TCrG, slow photocatalysis took place where the MB molecules were photodegraded by the generated free radicals.

### Antibacterial activity

**Figure 14:**
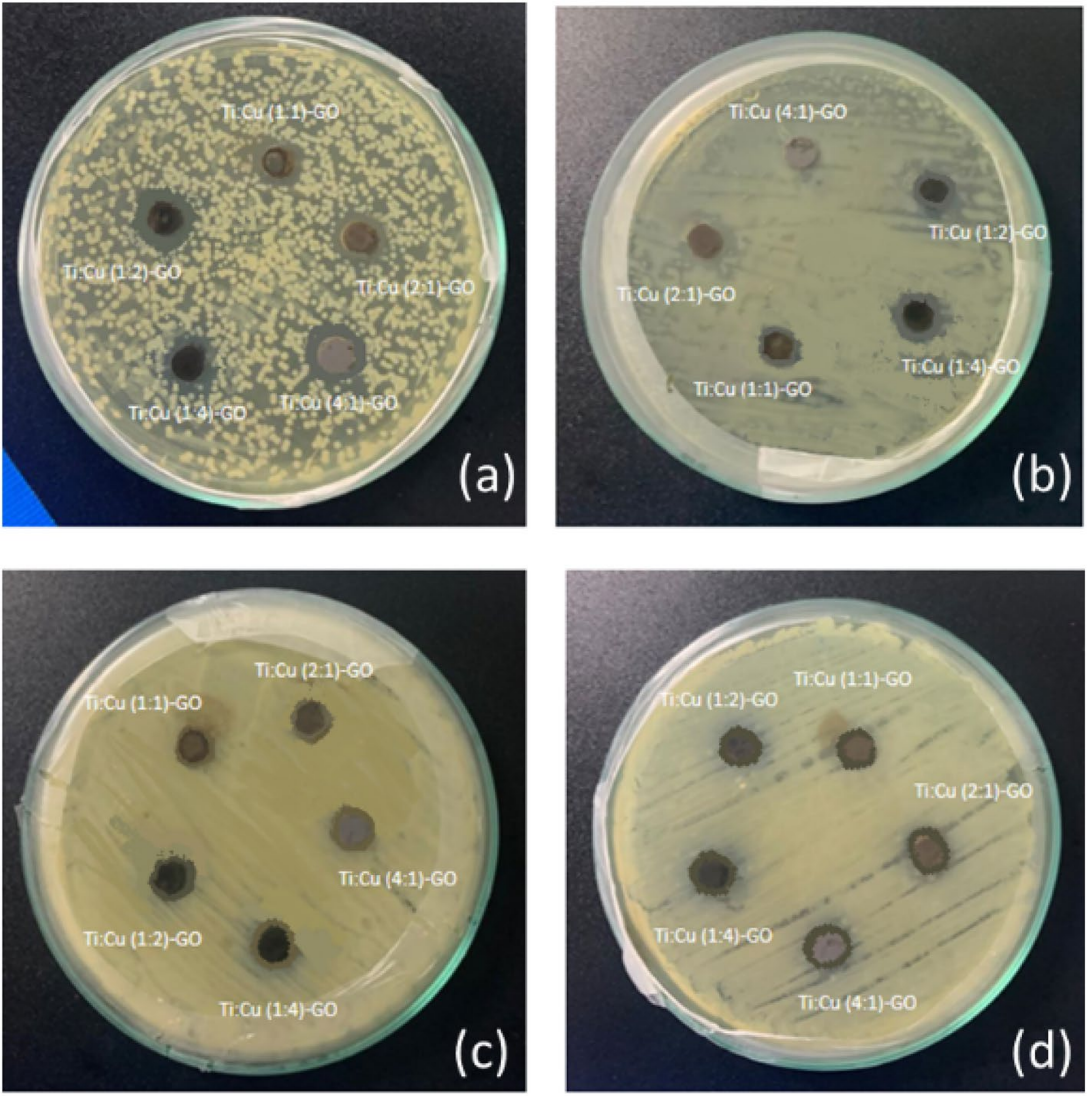
Antibacterial activity of synthesized nanomaterials (20 mg/ml) with test organisms. (**a**) *P. aeruginosa* and (**b**) *S. aureus* (**c**) *K. pneumonia* (**d**) *E. coli*.

Prior to their possible uses, it is essential to conduct a comprehensive assessment of the health and environmental consequences associated with nanomaterials. Metal oxides and Graphene exhibit potent cytotoxicity against microorganisms according to the published literature^52^. Much research has proposed potential pathways that include the interaction between nanomaterials, graphene-based compounds and biological molecules^53–58^. The results of the agar well diffusion method are shown in Fig. 14.

**Figure 15:**
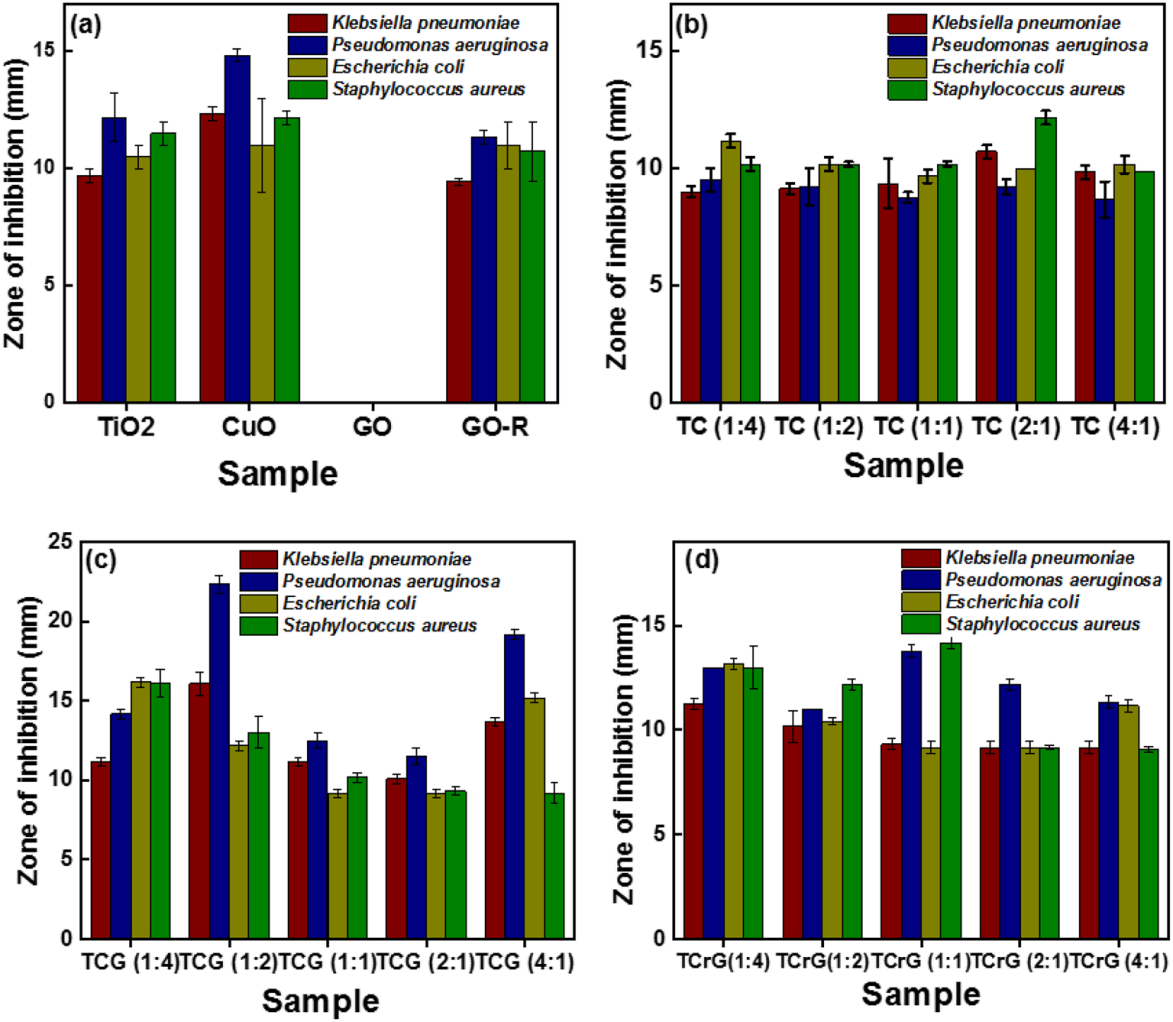
Zone of inhibitions of all the TC, TCG and TCrG composites against *E*.* coli*, *S*.* aureus*, *P*.* aeruginosa* and *K*.* pneumonia.*

Microorganisms are often described as possessing a negative charge, while metal oxides are known to contain a positive charge. This generates an “electromagnetic” force of attraction between the microorganism and the treated surface. Upon contact, the microorganism undergoes oxidation and immediately dies^56^. The antibacterial activity of the synthesized samples was evaluated against the growth of *E*.* coli*, *S*.* aureus*, *P*.* aeruginosa* and *K*.* pneumonia*. It was found that out of 19 samples tested, Ti:Cu (1:2)-GO and Ti:Cu (1:4)-GO exhibited the highest antibacterial activity against *K*.* pneumoniae* (16.08 ± 0.14 mm), *P*.* aeruginosa* (22.33 ± 0.58 mm), *E*.* coli* (16.17 ± 0.29 mm) and *S*.* aureus* (16.08 ± 0.88), respectively. Between the TiO_2_ and CuO, CuO alone indicated higher antibacterial activities against *K*.* pneumoniae* (12.33 ± 0.29 mm), *P*.* aeruginosa* (14.83 ± 0.29 mm), *E*.* coli* (11.00 ± 2.00 mm) and *S*.* aureus* (12.17 ± 0.29 mm), respectively (Fig. 15).

It is therefore clear that the larger dosage of CuO contributes to a greater inhibitory activity against pathogenic bacteria and it functions in a dose-dependent manner. Various mechanisms of antibacterial activity of CuO have been discussed in other studies. The CuO compound utilizes a contact killing mechanism that inflicts a lot of damage on the bacterial cell membrane. Subsequently, further damage occurs via a distinct mechanism involving the copper oxide nanoparticle. Toxicity to microorganisms is mostly caused by the production of free radicals from cupric oxide (CuO) since they generate a substantial amount of reactive oxygen species (ROS) in the form of superoxides^56^. Typically, bacterial cells are of a size that falls within the micrometer scale, while the outer cell membranes have holes that are represent the nanometer scale. Due to their smaller size compared to bacterial pores, CuO nanoparticles possess a distinct ability to penetrate the cell membrane^57,58^.

Titanium dioxide (TiO_2_) is another widely used metal oxide for the inhibition and sterilization of many types of microorganisms, such as bacteria, fungus, and viruses. This is due to its remarkable photoreactivity, broad-spectrum antibacterial properties, and chemical stability^59,60^. Priyanka et al*.* reported that TiO_2_ synthesized through sol gel method demonstrated antimicrobial activity against Gram-positive bacteria (*S*.* aureus*, *Streptococcus pneumoniae*, and *Bacillus subtilis*), Gram-negative bacteria (*Proteus vulgaris*, *P*.* aeruginosa*, and *E*.* coli*), and the fungal pathogen *Candida albicans* in daylight, and antimicrobial activity against *Streptococcus pneumonia*, *S*.* aureus*, *Proteus vulgaris*, *P*.* aeruginosa*, and *Candida albicans* in the visible light range^61^. The antibacterial activity of TiO_2_ synthesized through hybrid RF-sputtering/sol–gel approach, against *B*.* subtilis* and the respective mechanism has been demonstrated^62^. TiO_2_ nanotubes fabricated by electrochemical anodization demonstrated antibacterial activity against the pathogenic *E*.* coli*^63^.

The antibacterial efficacy of TiO_2_ nanoparticles (NPs) is due to the degradation of bacterial outer membranes caused by reactive oxygen species (ROS), specifically hydroxyl radicals (OH). This process results in the peroxidation of phospholipids and ultimately leads to the death of bacterial cells^64–66^. The strong oxidizing ability of TiO_2_ nanoparticles may be used to attack organic compounds present in bacterial cells^66,67^. Out of graphene oxide loaded samples and reduced graphene oxide loaded samples, the graphene oxide loaded samples showed higher antibacterial activity against the reduced graphene oxide loaded samples. Graphene oxide is essentially a type of graphene that has undergone oxidation, resulting in the addition of oxygen-based functional groups, for example hydroxyl (−OH), alkoxy (C–O–C), carbonyl, carboxylic acid (−COOH), and other oxygen-based functional groups on the sp_2_ carbon basal plane^53^. Graphene oxide contains epoxide functional groups on its basal plane and carboxylic functional groups on its edges, in addition to hydroxyl functional groups present on both the basal plane and the edges^68^.

Hence, graphene oxide has significant value in biological applications due to its notable characteristics such as hydrophilicity, excellent dispersion in water-based solutions, straightforward synthesis, strong structural stability, high compatibility with living organisms, minimal harmful effects on cells, and the ability to modify its surface through functional groups^69^. Furthermore, GO has the ability to interact with biomolecules, including proteins and nucleic acids. Some studies indicate that graphene or GO alone, either do not affect bacteria or instead stimulate the development of *E*.* coli* bacterium^70,71^. The antibacterial test results for GO are analogous to these findings, since they have no inhibitory impact on any pathogenic organism when tested alone.

In contrast, rGO is produced by the process of thermal annealing or chemical reduction of GO, resulting in a decrease in oxygenated functional groups^72^ and showed antibacterial activities when tested alone. Studies have mostly linked the antibacterial effect of rGO to the rupture of cells caused by the physical piercing of the cell membrane upon contact with the sharp edges of the particles^54,55^. Leakages of cytoplasmic contents and changes in the structure of the bacterial cell can be used to identify these forms of destruction. Moreover, the presence of sharp edges in rGO facilitates the generation of membrane stress, which physically harms the bacteria. Consequently, this leads to the cell membrane breaking down, followed by the release of RNA^55^. In a previous study which contrasted the antibacterial efficacy of GO and rGO, it was shown that both GO and rGO exhibited superior antibacterial properties compared to other compounds formed from graphene^73^.

**Figure 16:**
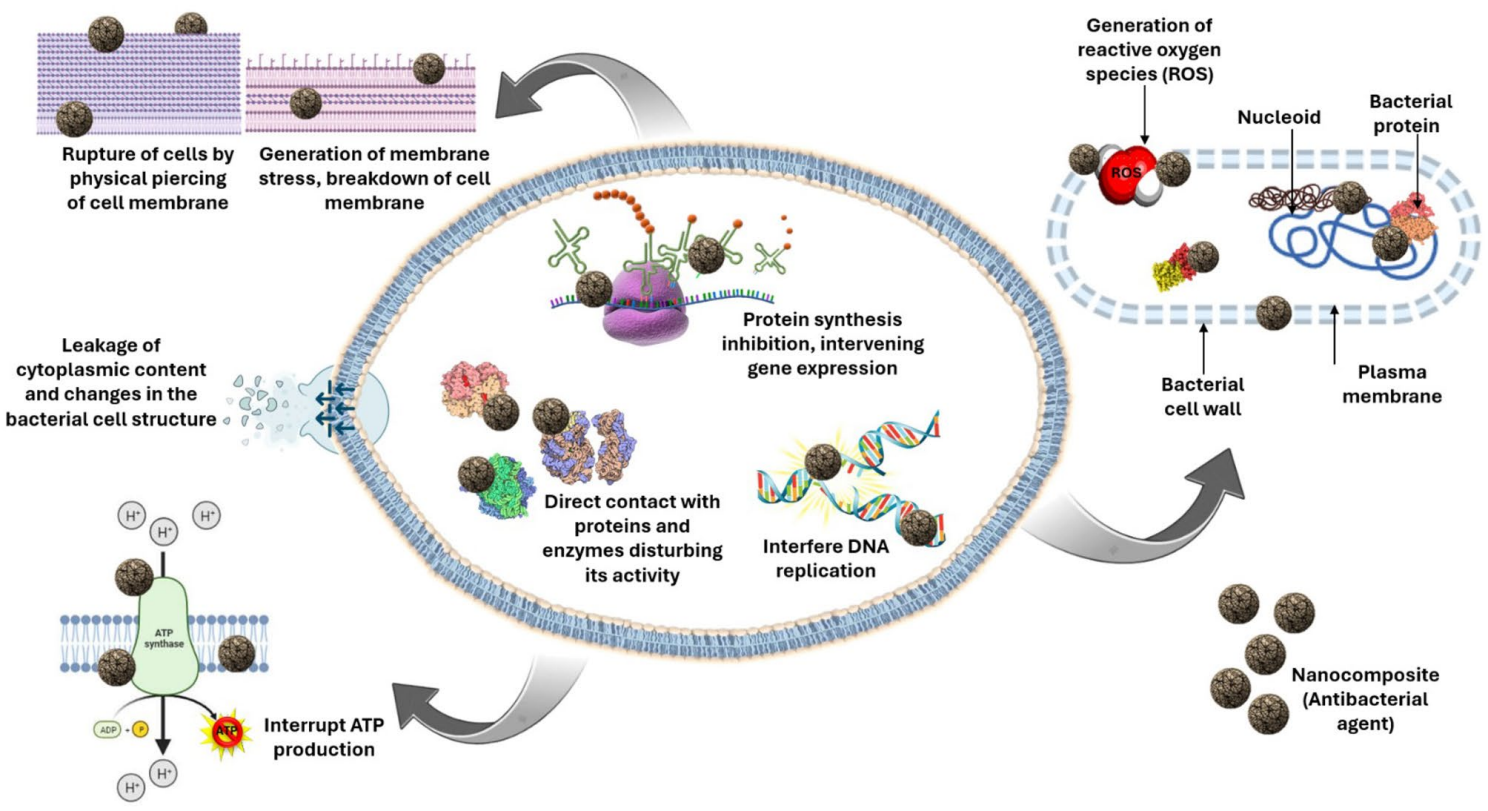
The proposed antibacterial mechanism.

The SEM image of GO in the current study shows the crumpled and wrinkled lamella structure obtained from the oxidation of graphite, and it can contribute to the antibacterial activity through direct contact. The edges of the new individual GO sheets in the wrinkled and crumpled structure may directly cause partial or complete penetration of the bacterial cells, which in turn may encourage inhibition of the bacteria. The presence of oxygenated groups on GO which results in the formation of wrinkles appearing at the edges and surface of the graphite flakes are due to oxidation. Moreover, the oxidation process is expanded to the carbon’s center, resulting in the addition of extra oxygenated groups between the layers. These oxygenated groups could possess a higher oxidation potential which may oxidize the biological molecules that could lead to the disruption of the cellular metabolism including nucleic acid synthesis, protein synthesis and even cell death. It has also been reported in the literature that both GO and rGO have the ability to cause the oxidation of glutathione, which functions as a mediator of the redox state in bacteria. Conductive rGO possesses greater oxidation capabilities compared to insulating GO^73^. The proposed antibacterial mechanism is shown in Fig. 16.

The findings strongly suggest that the antibacterial effects are guided by both membrane and oxidative stress. We propose a mechanism that involves the initial deposition of cells on test materials, the stress experienced by the membrane due to direct contact with sharp materials, and the subsequent oxidation of biomolecules. It is recommended that the physicochemical characteristics of graphene-based materials, including the functional groups, size, and conductivity, be deliberately modified, and optimized in order to enhance their potential for antibacterial applications.

## Conclusion

TiO_2_–CuO samples were fabricated hydrothermally in varying weight ratios, these being 1:4, 1:2, 1:1, 2:1 and 4:1. Then they were coupled with 30% GO hydrothermally and such coupled composited were reduced to produce TiO_2_–CuO /rGO successfully. TiO_2_–CuO and TiO_2_–CuO/GO composites effectively degraded MB in visible light whereas TiO_2_–CuO/rGO composites were effective in coagulating and flocculating MB and photocatalytically degrading MB as well in the presence of persulfate. The photocatalytic activity increased with increasing concentration of persulfate up to 4 mM in the best performing TC and TCG composites, these being TC (1:4) and TCG (1:1), respectively, and reduced when the concentration further rose to 8 mM. The rate constant increased 3.3-fold when the weight of the catalyst increased from 10 to 50 mg and fell to 5.3 × 10^–3^ min^−1^ upon increase to 100 mg in the presence of TC (1:4) while that increased 4.35-fold once the catalyst dosage increased from 10 to 100 mg. The rate constant only increases from 5 × 10^–2^ to 5.5 × 10^–2^ min^−1^ when the MB concentration increases from 5 to 10 mg/L in the presence of TC (1:4), which increased from 2.6 × 10^–2^ to 3.2 × 10^–2^ min^−1^ upon increase of the MB concentration from 5 to 20 mg/L.

Furthermore, photocatalytic activity waned with further increase in the MB concentration in both types of composites. EDTA and IPA reduce the photocatalytic activity scavenging holes and OH·, respectively. TCrG composites made possible the removal of MB by coagulation and flocculation in light and dark contexts, in the presence of persulfate ions while slow photodegradation was observed in light. All the TC, TCG and TCrG composites exhibited antibacterial activity that hindered the growth of *E*.* coli*, *S*.* aureus*, *P*.* aeruginosa* and *K*.* pneumonia*. Ti:Cu (1:2)-GO and Ti:Cu (1:4)-GO demonstrated the highest antibacterial activity against *P*.* aeruginosa* (22.33 ± 0.58 mm), *E*.* coli* (16.17 ± 0.29 mm), *S*.* aureus* (16.08 ± 0.88), and *K*.* pneumoniae* (16.08 ± 0.14 mm) out of the 19 samples tested. It is therefore evident that TiO_2_–CuO, TiO_2_–CuO/GO and TiO_2_–CuO/rGO composites are effective in removing MB and inhibiting the growth of bacteria in wastewater.

### Supplementary Information

Supplementary Information.

## Data Availability

The datasets used and/or analyzed during the current study are available from the corresponding author on reasonable request.

## References

[1] Przystas W, Zablocka-Godlewska E, Grabinska-Sota E (2012). Biological removal of azo and triphenylmethane dyes and toxicity of process by-products. Water. Air. Soil Pollut..

[2] Ben Mansour H (2012). Alteration of in vitro and acute in vivo toxicity of textile dyeing wastewater after chemical and biological remediation. Environ. Sci. Pollut. Res..

[3] Yaseen A, Yaseen DA, Scholz M (2018). Textile dye wastewater characteristics and constituents of synthetic effluents: A critical review. Int. J. Environ. Sci. Technol..

[4] Khatri A, Peerzada MH, Mohsin M, White M (2015). A review on developments in dyeing cotton fabrics with reactive dyes for reducing effluent pollution. J. Clean. Prod..

[5] Mendis A (2023). Fabrication of naturally derived chitosan and ilmenite sand-based TiO_2_/Fe_2_O_3_/Fe-N-doped graphitic carbon composite for photocatalytic degradation of methylene blue under sunlight. Molecules.

[6] Sarvajith M, Reddy GKK, Nancharaiah YV (2018). Textile dye biodecolourization and ammonium removal over nitrite in aerobic granular sludge sequencing batch reactors. J. Hazard. Mater..

[7] Suzuki M, Suzuki Y, Uzuka K, Kawase Y (2020). Biological treatment of non-biodegradable azo-dye enhanced by zero-valent iron (ZVI) pre-treatment. Chemosphere.

[8] Popli S, Patel UD (2015). Destruction of azo dyes by anaerobic–aerobic sequential biological treatment: a review. Int. J. Environ. Sci. Technol..

[9] de Amorim CC, Leão MMD, de Moreira RFPM (2009). Comparison of various advanced oxidation processes for azo dye degradation. Eng. Sanit. e Ambient.

[10] Ogugbue CJ, Sawidis T (2011). Bioremediation and detoxification of synthetic wastewater containing triarylmethane dyes by aeromonas hydrophila isolated from industrial effluent. Biotechnol. Res. Int..

[11] Rahman M, Rayhan M, Chowdhury M, Mohiuddin K, Chowdhury M (2018). Phytotoxic effect of synthetic dye effluents on seed germination and early growth of red amaranth. Fundam. Appl. Agric..

[12] Bhatia D, Sharma NR, Singh J, Kanwar RS (2017). Biological methods for textile dye removal from wastewater: A review. Crit. Rev. Environ. Sci. Technol..

[13] Liyanaarachchi H, Thambiliyagodage C, Lokuge H, Vigneswaran S (2023). Kinetics and thermodynamics study of methylene blue adsorption to sucrose- and urea-derived nitrogen-enriched, hierarchically porous carbon activated by KOH and H_3_PO_4_. ACS Omega.

[14] Gunathilaka, H., Thambiliyagodage, C., Usgodaarchchi, L. & Angappan, S. Effect of surfactants on morphology and textural parameters of silica nanoparticles derived from paddy husk and their efficient removal of methylene blue. In: *Proceedings of the International Conference on Innovations in Energy Engineering & Cleaner Production (IEECP’21)*, (2021).

[15] Gadekar MR, Ahammed MM (2016). Coagulation/flocculation process for dye removal using water treatment residuals: Modelling through artificial neural networks. Desalin. Water Treat..

[16] Karaboyaci M, Uǧur ŞS (2014). Ecological wool dyeing with pulps of lavender, broom, and red wine. J. Text. Inst..

[17] Liyanaarachchi H, Thambiliyagodage C, Liyanaarachchi C, Samarakoon U (2023). Efficient photocatalysis of Cu doped TiO_2_/g-C_3_N_4_ for the photodegradation of methylene blue. Arab. J. Chem..

[18] Pham VV (2022). S-Scheme α-Fe_2_O_3_/g-C_3_N_4_ nanocomposites as heterojunction photocatalysts for antibiotic degradation. ACS Appl. Nano Mater..

[19] Larumbe S, Monge M, Gómez-Polo C (2015). Comparative study of (N, Fe) doped TiO_2_ photocatalysts. Appl. Surf. Sci..

[20] Neppolian B, Sakthivel S, Arabindoo B, Palanichamy M, Murugesan V (1999). Degradation of textile dye by solar light using TiO_2_ and ZnO photocatalysts. J. Environ. Sci. Health Part A Toxic/Hazard. Subst. Environ. Eng..

[21] Liu Y (2014). Sandwich SrTiO_3_/TiO_2_/H-titanate nanofiber composite photocatalysts for efficient photocatalytic hydrogen evolution. Appl. Surf. Sci..

[22] Lai C (2019). Fabrication of CuS/BiVO_4_ (0 4 0) binary heterojunction photocatalysts with enhanced photocatalytic activity for Ciprofloxacin degradation and mechanism insight. Chem. Eng. J..

[23] Sabri M, Habibi-Yangjeh A, Rahim Pouran S, Wang C (2023). Titania-activated persulfate for environmental remediation: the-state-of-the-art. Catal. Rev. Sci. Eng..

[24] Gouvêa CAK (2000). Semiconductor-assisted photocatalytic degradation of reactive dyes in aqueous solution. Chemosphere.

[25] Reyes-Coronado D (2008). Phase-pure TiO_2_ nanoparticles: Anatase, brookite and rutile. Nanotechnology.

[26] Raizada P (2020). Engineering nanostructures of CuO-based photocatalysts for water treatment: Current progress and future challenges. Arab. J. Chem..

[27] Sun S, Zhang X, Cui J, Yang Q, Liang S (2019). Tuning interfacial Cu-O atomic structures for enhanced catalytic applications. Chem. Asian J..

[28] Borowska E (2019). Solar photocatalytic degradation of sulfamethoxazole by TiO_2_ modified with noble metals. Catalysts.

[29] Ismael M (2020). Enhanced photocatalytic hydrogen production and degradation of organic pollutants from Fe (III) doped TiO_2_ nanoparticles. J. Environ. Chem. Eng..

[30] Anil Kumar Reddy P (2010). Photocatalytic degradation of isoproturon pesticide on C, N and S Doped TiO_2_. J. Water Resour. Prot..

[31] Jaiswal R (2015). Copper and nitrogen co-doped TiO_2_ photocatalyst with enhanced optical absorption and catalytic activity. Appl. Catal. B Environ..

[32] Khan MI (2018). Synthesis, characterization and application of Co doped TiO_2_ multilayer thin films. Results Phys..

[33] Bessegato GG, Cardoso JC, Zanoni MVB (2015). Enhanced photoelectrocatalytic degradation of an acid dye with boron-doped TiO_2_ nanotube anodes. Catal. Today.

[34] Sher M, Shahid S, Javed M (2021). Synthesis of a novel ternary (g-C3N4 nanosheets loaded with Mo doped ZnOnanoparticles) nanocomposite for superior photocatalytic and antibacterial applications. J. Photochem. Photobiol. B Biol..

[35] Qamar MA, Javed M, Shahid S, Sher M (2022). Fabrication of g-C_3_N_4_/transition metal (Fe Co, Ni, Mn and Cr)-doped ZnO ternary composites: Excellent visible light active photocatalysts for the degradation of organic pollutants from wastewater. Mater. Res. Bull..

[36] Sher M (2021). The controlled synthesis of g-C_3_N_4_/Cd-doped ZnO nanocomposites as potential photocatalysts for the disinfection and degradation of organic pollutants under visible light irradiation. RSC Adv..

[37] Chen P, Li J, Wang J, Deng L (2024). Synergistic enhancement of carrier migration by SnO_2_/ZnO@GO heterojunction for rapid degradation of RhB. Molecules.

[38] Mohamed A, Mahanna H, Samy M (2024). Synergistic effects of photocatalysis-periodate activation system for the degradation of emerging pollutants using GO/MgO nanohybrid. J. Environ. Chem. Eng..

[39] Balouiri M, Sadiki M, Ibnsouda SK (2016). Methods for in vitro evaluating antimicrobial activity: A review. J. Pharm. Anal..

[40] Thambiliyagodage C, Nakandala S, Siriwardana B, Lansakara B (2021). One pot synthesis of α-Fe_2_O_3_/turbostratic carbon composites and their photocatalytic activity under sunlight. Carbon Trends.

[41] Ganesh S, Thambiliyagodage C, Perera SVTJ, Rajapakse RKND (2023). Influence of laboratory synthesized graphene oxide on the morphology and properties of cement mortar. Nanomaterials.

[42] Dac Dien N (2019). Preparation of various morphologies of ZnO nanostructure through wet chemical methods. Adv. Mater. Sci..

[43] Sayahi H, Aghapoor K, Mohsenzadeh F, Mohebi Morad M, Darabi HR (2021). TiO_2_ nanorods integrated with titania nanoparticles: Large specific surface area 1D nanostructures for improved efficiency of dye-sensitized solar cells (DSSCs). Sol. Energy.

[44] Thambiliyagodage C (2022). Efficient visible-light photocatalysis and antibacterial activity of TiO_2_-Fe_3_C-Fe-Fe_3_O_4_/graphitic carbon composites fabricated by catalytic graphitization of sucrose using natural ilmenite. ACS Omega.

[45] Usgodaarachchi L, Jayanetti M, Thambiliyagodage C, Liyanaarachchi H, Vigneswaran S (2022). Fabrication of r-GO/GO/α-Fe_2_O_3_/Fe_2_TiO_5_ nanocomposite using natural ilmenite and graphite for efficient photocatalysis in visible light. Materials.

[46] Peng WC (2018). Tunability of p- and n-channel TiOx thin film transistors. Sci. Rep..

[47] Wang Y (2015). Synthesis of porous Cu_2_O/CuO cages using Cu-based metal–organic frameworks as templates and their gas-sensing properties. J. Mater. Chem. A.

[48] Li Q, Xie R, Yin WL, Mintz EA, Jian KS (2007). Enhanced visible-light-induced photocatalytic disinfection of E. coli by carbon-sensitized nitrogen-doped titanium oxide. Environ. Sci. Technol..

[49] Singh M, Goyal M, Devlal K (2018). Size and shape effects on the band gap of semiconductor compound nanomaterials. J. Taibah Univ. Sci..

[50] Zhong X, Zou Z-S, Wang H-L, Huang W, Zhou B-X (2019). Enhanced activation of persulfate by Co-doped bismuth ferrite nanocomposites for degradation of levofloxacin under visible light irradiation. Materials.

[51] Zhang T (2020). Enhanced photocatalytic activity of TiO_2_ with acetylene black and persulfate for degradation of tetracycline hydrochloride under visible light. Chem. Eng. J..

[52] Stanić V, Tanasković SB (2020). Antibacterial activity of metal oxide nanoparticles. Nanotoxicity prevention and antibacterial applications of nanomaterials.

[53] Ghulam AN (2022). Graphene oxide (GO) materials—applications and toxicity on living organisms and environment. J. Funct. Biomater..

[54] Hu W (2010). Graphene-based antibacterial paper. ACS Nano.

[55] Sengupta I (2019). Bactericidal effect of graphene oxide and reduced graphene oxide: Influence of shape of bacteria. Colloid Interface Sci. Commun..

[56] Khashan KS (2021). Antibacterial activity of TiO_2_ nanoparticles prepared by one-step laser ablation in liquid. Appl. Sci..

[57] Fahmy B, Cormier SA (2009). Copper oxide nanoparticles induce oxidative stress and cytotoxicity in airway epithelial cells. Toxicol. Vitr..

[58] Dadi R, Azouani R, Traore M, Mielcarek C, Kanaev A (2019). Antibacterial activity of ZnO and CuO nanoparticles against gram positive and gram negative strains. Mater. Sci. Eng. C.

[59] Nadtochenko V (2006). Laser kinetic spectroscopy of the interfacial charge transfer between membrane cell walls of E. coli and TiO_2_. J. Photochem. Photobiol. A Chem..

[60] Rincón AG, Pulgarin C (2004). Effect of pH, inorganic ions, organic matter and H2O2 on E. coli K12 photocatalytic inactivation by TiO_2_: Implications in solar water disinfection. Appl. Catal. B Environ..

[61] Priyanka KP, Sukirtha TH, Balakrishna KM, Varghese T (2016). Microbicidal activity of TiO_2_ nanoparticles synthesised by sol–gel method. IET Nanobiotechnol..

[62] Armelao L (2007). Photocatalytic and antibacterial activity of TiO_2_ and Au/TiO_2_ nanosystems. Nanotechnology.

[63] Li H (2013). Antibacterial activity of TiO_2_ nanotubes: Influence of crystal phase, morphology and Ag deposition. Appl. Surf. Sci..

[64] Shah MSAS, Nag M, Kalagara T, Singh S, Manorama SV (2008). Silver on PEG-PU-TiO_2_ polymer nanocomposite films: An excellent system for antibacterial applications. Chem. Mater..

[65] Ishibashi KI, Fujishima A, Watanabe T, Hashimoto K (2000). Generation and deactivation processes of superoxide formed on TiO_2_ film illuminated by very weak UV light in air or water. J. Phys. Chem. B.

[66] Cho KH, Park JE, Osaka T, Park SG (2005). The study of antimicrobial activity and preservative effects of nanosilver ingredient. Electrochim. Acta.

[67] Shiraishi Y, Hirai T (2008). Selective organic transformations on titanium oxide-based photocatalysts. J. Photochem. Photobiol. C Photochem. Rev..

[68] Compton OC, Nguyen ST (2010). Graphene oxide, highly reduced graphene oxide, and graphene: Versatile building blocks for carbon-based materials. Small.

[69] Smith AT, LaChance AM, Zeng S, Liu B, Sun L (2019). Synthesis, properties, and applications of graphene oxide/reduced graphene oxide and their nanocomposites. Nano Mater. Sci..

[70] Chen H, Müller MB, Gilmore KJ, Wallace GG, Li D (2008). Mechanically strong, electrically conductive, and biocompatible graphene paper. Adv. Mater..

[71] Park S (2010). Biocompatible, robust free-standing paper composed of a TWEEN/graphene composite. Adv. Mater..

[72] Yun JH, Ng YH, Wong RJ, Amal R (2013). Reduced graphene oxide: control of water miscibility, conductivity, and defects by photocatalysis. ChemCatChem.

[73] Liu S (2011). Antibacterial activity of graphite, graphite oxide, graphene oxide, and reduced graphene oxide: Membrane and oxidative stress. ACS Nano.

