# Supplementary material for: Persulfate assisted photocatalytic and antibacterial activity of TiO2–CuO coupled with graphene oxide and reduced graphene oxide
Source: Sci Rep. 2024 May 31;14:12505. doi: 10.1038/s41598-024-63452-7 (PMC11143361; doi:10.1038/s41598-024-63452-7)
Supplement: Supplementary file 1 — Supplementary Information. [file 41598_2024_63452_MOESM1_ESM.docx]

**Supplementary Information**

**Persulfate assisted photocatalytic and antibacterial activity of TiO2/CuO coupled with graphene oxide and reduced graphene oxide**

Charitha Thambiliyagodage^1,*^, Heshan Liyanaarachchi^1^, Madara Jayanetti^1^, Geethma Ekanayake^1^, Amavin Mendis^1^, Upeka Samarakoon^2^, Saravanamuthu Vigneswaran^3,4^

^1^ Faculty of Humanities and Sciences, Sri Lanka Institute of Information Technology, Malabe, Sri Lanka

^2^ Department of Nano Science Technology, Faculty of Technology, Wayamba University of Sri Lanka, Kuliyapitiya, Sri Lanka

^3^ Faculty of Engineering and Information Technology, University of Technology Sydney, PO Box 123, Broadway, NSW 2007, Australia

^4^ Faculty of Sciences & Technology (RealTek), Norwegian University of Life Sciences, P.O. Box N-1432 Ås, Norway

*[charitha.t@sliit.lk](about:blank)


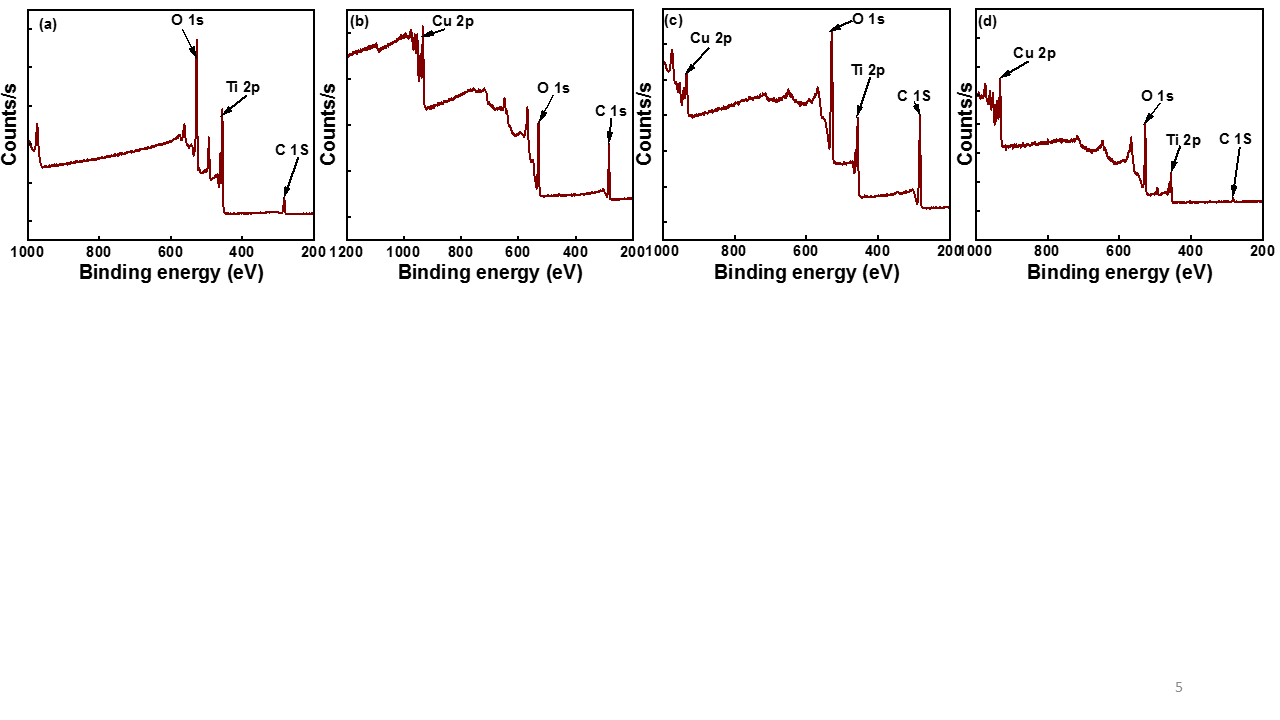


Figure 1. Survey spectra of (a) TiO2 (b) CuO (c) TC (1:1) (d)TCG (1:1)


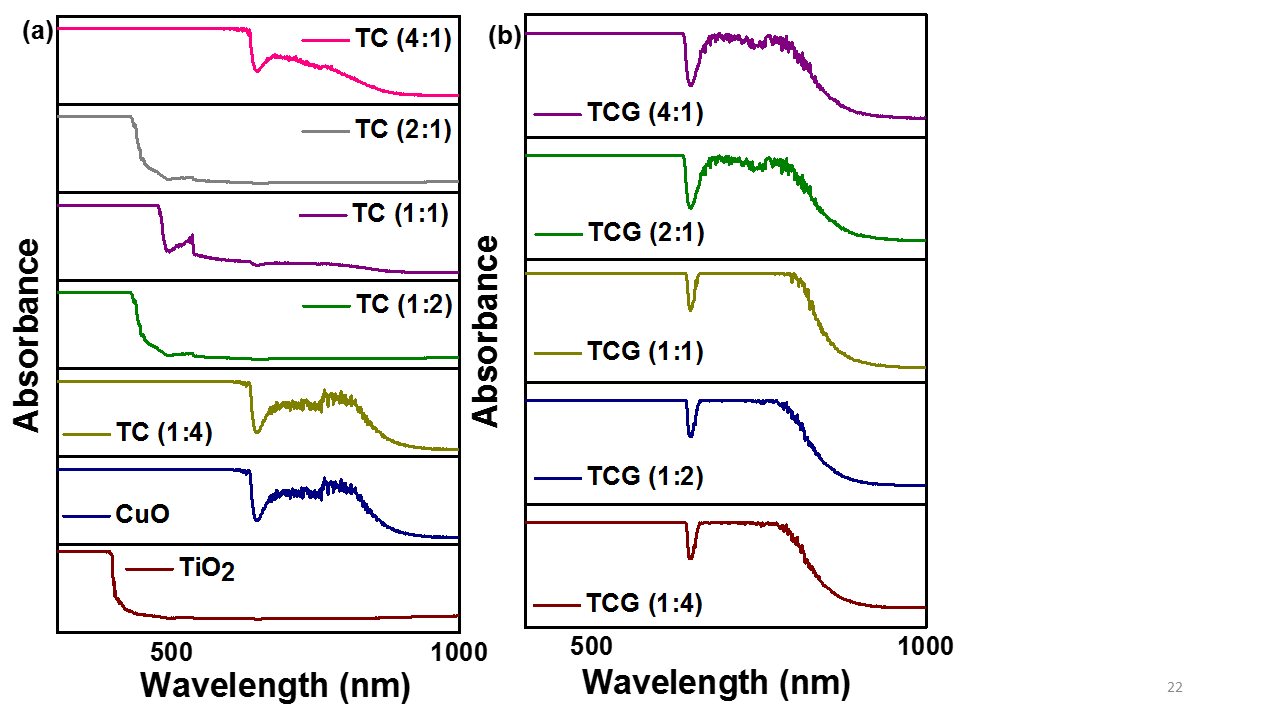


Figure 2. UV-Visible absorption spectra of the synthesized materials


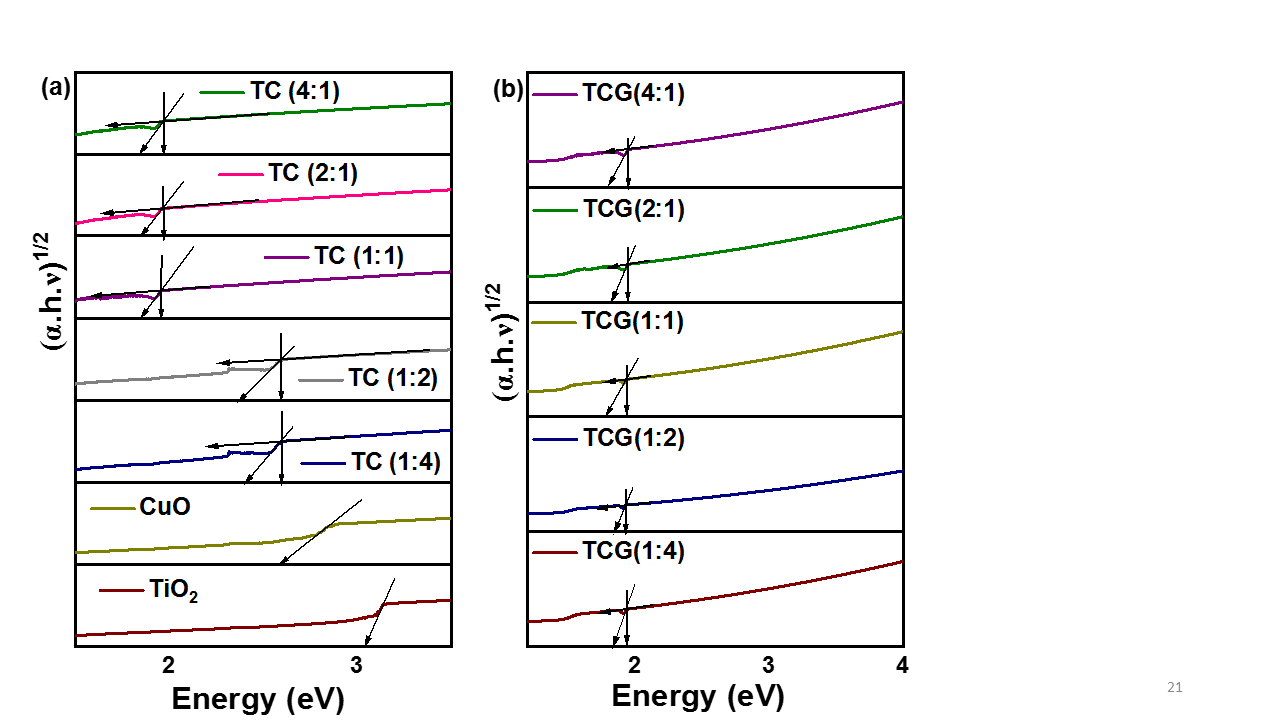


Figure 3. Tauc plots of direct transitions
